# Positive selection for the male functionality of a co-retroposed gene in the hominoids

**DOI:** 10.1186/1471-2148-9-252

**Published:** 2009-10-15

**Authors:** Yong Zhang, Shujuan Lu, Shuqi Zhao, Xiaofeng Zheng, Manyuan Long, Liping Wei

**Affiliations:** 1 Center for Bioinformatics, National Laboratory of Protein Engineering and Plant Genetic Engineering, College of Life Sciences, Peking University, Beijing, 100871, PR China; 2 Department of Ecology and Evolution, the University of Chicago, 1101 E 57 Street, Chicago, IL 60637, USA

## Abstract

**Background:**

New genes generated by retroposition are widespread in humans and other mammalian species. Usually, this process copies a single parental gene and inserts it into a distant genomic location. However, retroposition of two adjacent parental genes, *i.e*. co-retroposition, had not been reported until the hominoid chimeric gene, *PIPSL*, was identified recently. It was shown how two genes linked in tandem (phosphatidylinositol-4-phosphate 5-kinase, type I, alpha, *PIP5K1A *and proteasome 26S subunit, non-ATPase, 4, *PSMD4*) could be co-retroposed from a single RNA molecule to form this novel chimeric gene. However, understanding of the origination and biological function of *PIPSL *requires determination of the coding potential of this gene as well as the evolutionary forces acting on its hominoid copies.

**Results:**

We tackled these problems by analyzing the evolutionary signature in both within-species variation and between species divergence in the sequence and structure of the gene. We revealed a significant evolutionary signature: the coding region has significantly lower sequence variation, especially insertions and deletions, suggesting that the human copy may encode a protein. Moreover, a survey across five different hominoid species revealed that all adaptive changes of *PSMD4*-derived regions occurred on branches leading to human and chimp rather than other hominoid lineages. Finally, computational analysis suggests testis-specific transcription of *PIPSL *is regulated by tissue-dependent methylation rather than some transcriptional leakage.

**Conclusion:**

Therefore, this set of analyses showed that *PIPSL *is an extraordinary co-retroposed protein-coding gene that may participate in the male functions of humans and its close relatives.

## Background

Retroposition, an RNA-intermediated copy mechanism, could shape genomes widely in eukaryotes, and in particular plays a substantial role in evolution of functional novelties [1]. People ever viewed it as a trivial molecular process for making functionless processed pseudogenes [2]. However, extensive analyses have revealed that a large number of retrosequences have acquired various functions from vertebrates to invertebrates [3,4], from spermatogenesis [3] to courtship behaviors [5]. Many retrogenes could recruit nearby preexisting exon-intron sequences and genomic regions to form a chimeric gene structure with novel protein structures, which may expand protein functional diversity [6-8].

Almost all observed retroposition events involve a single parental gene that serves as substrate for retroposition. However, Akiva *et al *recently observed an extraordinary case that two adjacent genes in the hominoid lineage, *PIP5K1A *and *PSMD4 *in chromosome 1, co-retroposed from a read-through transcript and formed a new chimeric gene, *PIPSL*, in chromosome 10 [9]. PIP5K1A encodes the alpha isoform of phosphatidylinositol 4-phosphate 5-kinase type I (PIP5K), which is involved in the synthesis of two essential second messengers, 1,2-diacylglycerol and inositol 1,4,5-trisphosphate [10,11]. On the other hand, PSMD4 recruits ubiquitylated substrates to the proteasome for their degradation, which is mediated by two conserved 20-30 residual hydrophobic regions, *i.e*., ubiquitin-interacting motifs (UIMs) [12].

Northern analyses revealed high expression of *PIPSL *in testis in humans and chimpanzee and base-level or undetectable expression in other tissues [13]. The human *PIPSL *possesses a 1 bp in-frame deletion at +45 bp with respect to the start codon, which is fixed in human populations. Such a deletion causes an early frameshift with disrupted translation such that western blotting does not reveal protein product in human. However, Western blotting also fails to detect proteins in chimpanzee which does not have such a deletion. Therefore, *PIPSL *was believed to undergo translational silencing in both species [13]. Comparison of the substitution rates between synonymous and nonsynonymous rates revealed that the C-terminal portion of *PIPSL *derived from *PSMD4 *possibly undergoes positive selection in the early stage of the hominoid clade while the *PIP5K1A*-derived portion of the N-terminal portion in later stage of the hominoid clade does not depart from neutral expectation [13]. The authors even propose *PIPSL *might be detrimental in human population now.

These analyses provided valuable data to understand the origination process and the characteristics of *PIPSL*. Moreover, this extraordinary gene raised two interesting questions for further pursuit. First, considering no protein product is detected, is *PIPSL *a functional protein-coding gene or it is a processed pseudogene that has never had or has lost its function in human? Second, there were only two species of hominoid species, *i.e*., human and chimp in the analysis. Such limited dataset renders it hard to differentiate between the following two scenarios: positive selection indeed occurred on evolutionary branches toward human/chimp; positive selection occurs on branches leading to other hominoids, *e.g*., orangutan and gibbon and thus signal observed by comparing human *PIPSL *and its two parental genes is a by-product. If the later case is true, it will be reasonable to expect the repressed translation in both human and chimp since *PIPSL *might be only functional in orangutan and gibbon rather than human and chimp.

We tackled these problems by analyzing the evolutionary signature relating to the coding potential of *PIPSL *in both within-species variation and between species divergence. Such a strategy, complementary to the biological analyses of Babushock *et al*. (2007) and Akiva *et al*. (2006), revealed that the present-day *PIPSL *is subject to significant evolutionary constraint that shapes the standing variation in natural populations of the functional protein coding gene in humans and maintains its open reading frame (ORF). More than that, comparative analysis reveals that the adaptive evolution occurred in the lineage toward human and chimpanzee. Finally, genome-wide analysis of retro-pseudogenes suggests testis-specific transcription of *PIPSL *is not due to transcriptional leakage but highly regulated by tissue-dependent methylation. These analyses, in conjunction of previous expression data at RNA level, suggest that *PIPSL *is a functional retro-fusion testis-specific gene. Thus, the extraordinary co-retroposition mechanism played a role in the evolution of the male-specific functions in the lineage toward the humans.

## Results

### The lower nucleotide polymorphism and high structural integrity suggest *PIPSL *has coding potential

#### (1) CDS has a lower polymorphism level

**Figure 1:**
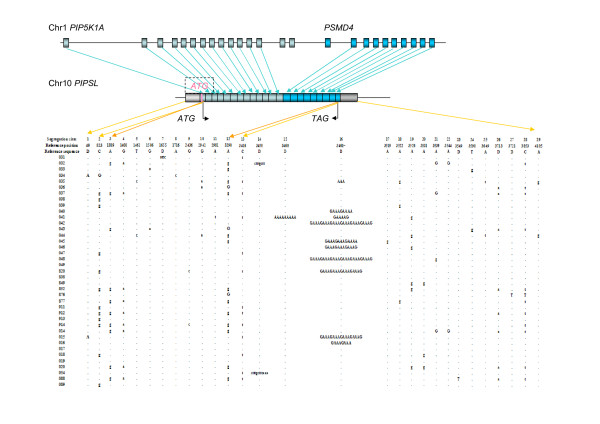
**Gene structure of *PIPSL *and its polymorphisms**. The aqua arrows between top two bars mark the correspondence between two parental genes and *PIPSL*. "ATG" and "TAG" in black indicate the border of the ORF. It is almost the complete fusion product of *PIP5K1A *and *PSMD4*, although the original start codon (the boxed "ATG" in rose) was destroyed due to a human specific deletion [13]. The distance between this original start codon and the current assumed start codon is only 60 bps. The left-hand and right-hand pale blocks mark the sequenced promoter region and 3' UTR region, respectively. The gold arrows indicate from which region polymorphisms are, like 5' UTR, coding region and 3' UTR. "Segregating sites" show the ID of polymorphisms. "Reference position" indicates the position relative to the starting point of sequenced reads. The first base corresponds to 592 bp upstream relative to the transcription start site of *PIPSL*. "Reference sequence" marks the consensus sequence in those locations with "D" indicating deletions relative to the consensus, with the nucleotides deleted shown in individuals. Letters in uppercase indicate homozygous mutations, while letters in lowercase indicate heterozygous mutations. "031","032" and so on indicate ID of samples. Herein, 031~040, 041~049, 820~914 and 014~089 are samples from African American, Africans in the south of the Sahara, Russian and Chinese, respectively.

The summary statistics for the population genetics of *PIPSL *are shown in Table 1 and Figure 1 (see also Additional file 1). First, our polymorphism data suggests 5' UTR and 3' UTR might undergo different selection force. Table 1 shows the high constraint of 5' flanking region with a nucleotide diversity (*π*) of 4 × 10^-4^, which might correspond to a functional role. Consistently, the published Chip-chip experiment of the Ludwig institute [14] shows this small region is highly enriched with transcription factor binding sites (see Additional file 2).

Moreover, as expected, CDS has fewer polymorphisms in terms of *π *of 4 × 10^-4 ^compared to the genome-wide background, 1 × 10^-3^[15]. It also has lower polymorphism level in terms of SNPs and indels compared to 3' flanking region. In terms of selection coefficient, *θ*, it is three fold lower in that there are 11 SNPs across 875 bp 3' flanking region, while there are only nine SNPs across 2,589 bp CDS. We tested whether or not this difference departs from neutral assumptions using Hudson's formula [16]. In this case, *l*, *n *and *s *are 2,589, 78 and 9, respectively. As a result, the polymorphism level of CDS is significantly lower compared to 3' UTR if the whole locus is homogeneously neutral (Table 2).

**Table 1:** Statistics of polymorphism, which was generated by DnaSP [39].

**Locus^A^**	**Length (bp)**	**#Single nucleotide polymorphisms**	***θ*/site^B^**	***π*/site**	**#indels**	***π*^*indel*^/site^D^**
complete	4,200	21	0.00101	0.00064	8	0.0002
5'flanking	736	1	0.00028	0.00041	1	0.0001
3'flanking	875	11	0.00255	0.00148	6	0.0010
*PIPSL*-CDS	2,589	9	0.00071	0.00042	1^C^	0.00001

A. "Complete" indicates the full locus of *PIPSL*, which consists of 736 bp 5' flanking region, 1,482 bp *PIP5K1A*-derived CDS, 1,107 bp *PSMD4*-derived CDS and 875 bp 3' flanking region. 5' flanking region includes both 592 bp promoter and 144 bp 5' UTR, while 3' flanking region indicates 3' UTR.

B. *θ *is the selection coefficient or 4*N*_*e*_*μ *(*N*_*e *_and *μ *indicates the effective population size and mutation rate, respectively).

C. This unique indel is heterozygous.

D. Indel diversity is calculated using the most conservative way, i.e., we only count diallelic difference and regard the copy number variants in 3' UTR as only two alleles. Thus, the indel diversity of 3' UTR might be underestimated.

**Table 2:** The probability of CDS generating not more than nine SNPs if the whole *PIPSL *locus is homogeneously neutral.

**Locus**	**Length (bp)**	***S***_*obs*_	**θ/Site**	***P *(*S*<=*S*_*obs*_|*θ *= 0.00255)**
*PIPSL*-CDS	2,589	9	0.00071	0.0005
3'flanking	875	11	0.00255	~

However, can this result be accounted for by a difference in local mutation bias, *e.g*., 3' UTR has a higher mutation rate? If so, we expect that 3' UTR would have a higher between-species divergence as well. However, we only observed nine substitutions in 3' UTR compared to 31 substitutions in CDS (Table 3). By comparing polymorphism and divergence, HKA test shows CDS significantly departs from 3' UTR (*p *= 0.03, Table 3) with possible excess of substitutions in 3'UTR or the decrease of substitutions in CDS. In order to disentangle both possibilities, we surveyed the polymorphism data of the *PIPSL *locus in gibbon. According to the trace data, this locus seems pseudogenic, which could serve as a neutral background. Out of two chromosomes from one female gibbon, nine segregating sites were identified, three derived from 3'UTR and six derived from CDS. Given selection coefficient, *θ*, the expected number of segregating sites, *S*, follows this formula [17]:

**Table 3:** HKA test using chimp as the outgroup.

**Locus**	**Length (bp)**	**Human polymorphisms**		**Human/chimp Divergences**		**Chi-square test**
		***S*_*obs*_**	***S*_*exp*_**	**#_*obs*_**	**#_*exp*_**	
			
*PIPSL*-CDS	2,589	9	13.33	31	26.67	
3'flanking	875	11	6.67	9	13.33	*p *= 0.0343



where "*n*" is 78 and 2 for our case and the gibbon genome sequence, respectively. In other words, if we increase the sample size of gibbon sequences to 78, the expected number of segregating sites in 3' UTR and CDS should be about 10 and 20, respectively. It is notable this iterative formula usually require multiple alleles like six or even more. Therefore, the estimation of 10 or 20 might not be that accurate. However, such a small sample does show CDS might have more polymorphisms than that of 3' UTR in gibbon *PIPSL *locus. Therefore, considering the observed data in human, 11 substitutions in 3'UTR and 9 substitutions in CDS, the deviation of human polymorphism data from the neutral expectation should be more likely attributed to the increasing constraint in CDS region.

#### (2) Both polymorphism data and evolutionary simulation suggests maintenance of *PIPSL*'s ORF is not a by-chance event

The polymorphism data of Table 1 also show that CDS appears to avoid indels compared to both flanking regions. Specifically, there are five homozygous indels and two heterozygous indels across 1.6 kb flanking regions; by contrast, there is only one heterozygous indel across 2,589 bp coding region (Fisher Exact Test *p *< 10^-4^). Regarding indel diversity, it is an order-of-multitude larger in UTR (6 × 10^-4^) than in CDS (1 × 10^-5^). Based on Hudson's formula [16], the probability to observe only one indel in an CDS of 2,589 bp is only 0.001 if CDS has as identical indel coefficient as that of UTR.

Using between-species data and by performing forward-simulation from the ancient sequence of *PIPSL *in hominoid, we further tested how it might be possible to maintain one ORF of 2,589 bp. Specifically, taking advantage of NCBI trace data, we assembled the complete *PIPSL *locus in orangutan and gibbon given the high read coverage (>4x) around this locus. By contrast, we were only able to assemble *PIP5K1A*-derived region in gorilla due to its lower sequencing coverage in *PSMD4*-derived region. Like human and chimp, orangutan maintains its *PIPSL *ORF. However, the gibbon *PIPSL *lost its coding potential by accumulating three nonsense substitutions and one in-frame indel (See Additional file 3). Gorilla seems to lie in a similar case with one nonsense substitution (CGA->TGA) in the middle of *PIP5K1A*-derived region.

We implemented ReEVOLVER [18] and forward simulated emergences of nonsense substitutions or frame-shifting indels together with nonsynonymous mutations. As a result, two tests of ReEVOLVER support the functionality of *PIPSL *(Table 4). Specifically, in the case of the complete sequence, *PIPSL *demonstrates high constraint revealed by both small Na/Ns (the ratio between number of nonsynonymous substitutions and that of synonymous substitutions) ratio (*P*_NaNs_~0.01) and frame-disrupting features (stop codons or frame shifts) (*P*_dis _< 10^-5^) compared to the expectation under neutrality. Furthermore, if we analyze *PSMD4*-derived region and *PIP5K1A*-derived region separately, both of them are also constrained as shown by highly significant *P*_dis_. As complementary evidence, we also investigated the process by estimating the time necessary to disrupt *PIPSL*'s ORF [19]. The time is only 1.9 million years and thus the probability to maintain this ORF is 1 × 10^-3 ^(See also Methods), which again suggests its protein-level constraint.

**Table 4:** 100,000 simulations are ran to track frame-disrupting features in case of CDS region derived from *PSMD4*, CDS region derived from *PIP5K1A *and the complete CDS of *PIPSL*, respectively.

**Locus**	**Ancestor Reconstruction^A^**	**Na/Ns_*obs*_**	***P*_NaNs_^B^**	**#Nonsense_*obs*_**	**#indels_*obs*_**	***P*_dis_^C^**
*PSMD4*	PAML	2.53	0.417	1	1	0.00071^D^
	Dnapars		0.402			0.00093
*PIP5K1A*	PAML	1.50	0.00045	2	0	<10^-5^
	Dnapars		0.00044			<10^-5^
Complete-Gene	PAML	1.91	0.01016	3	1	<10^-5^
	Dnapars		0.00879			<10^-5^

A. We used two distinct methods to reconstruct the ancestral sequence, PAML and Dnapars of Phylip package (Maximum-parsimony based inference). In all cases, there results are similar between each other.

B. *P*_NaNs _is defined as the proportion of simulated datasets that show a Na/Ns ratio smaller or equal to the observation. Here, Na and Ns indicates the number of nonsynonymous mutations and that of synonymous mutations, respetively.

C. *P*_dis _corresponds to the percentage of simulated datasets that demonstrates a number of frame-disrupting mutations (stop codons and frameshifts) smaller or equal to the observed number. Out of all lineages of interest, human, chimp, orangutan and gibbon, only three stop codons and one indel are observed and those only in the gibbon genome. Specifically, two nonsense substitutions occur in the *PIP5K1A*-derived region, while the other one nonsense substitution and one indel situate in the *PSMD4*-derived region.

### Positive selection of *PSMD4*-derived region only leads to human/Chimpanze lineage

The above analysis reveals that natural selection maintained a long ORF from the split of human and gibbon at 18 million years ago (Mya). Taking advantage of recently available sequence data of gibbon, orangutan and gorilla, we investigated this process with higher resolution. We used CODEML of PAML package [20] to infer the background selection force (See also Method section).

#### (1) Heterogeneous selection in different species

**Figure 2:**
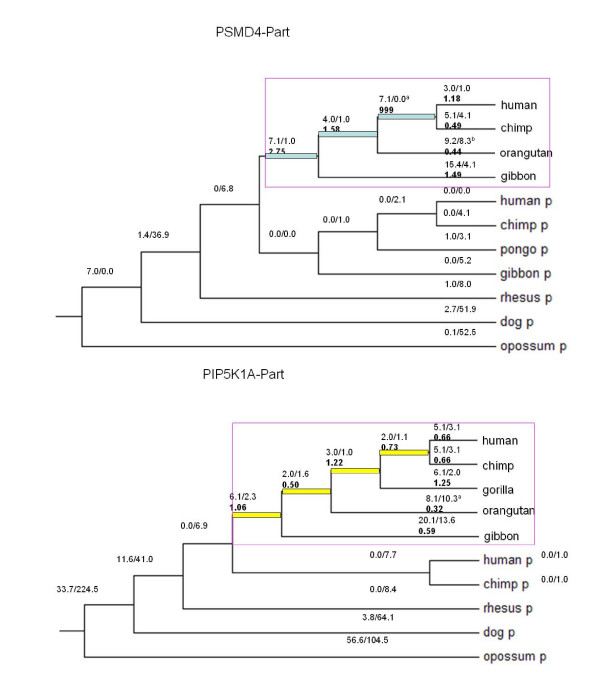
**The evolutionary process of *PSMD4*-derived region (top panel) and *PIP5K1A*-derived region (bottom panel) inferred based on the free ratio model of CODEML**. Blue and yellow bars marks ancestral branches leading to human and chimp in *PSMD4*-derived region and *PIP5K1A*-derived region, respectively. "P" indicates the parental gene. The number like "5.1/3.1" indicates how many nonsynonymous substitutions and synonymous substitutions occur in this branch, while the number in thicker font like "0.66" indicates *Ka/Ks*. In addition, we mark all branches with *Ka/Ks *significantly different with one by "a", which means a *p *of 0~0.05. Considering the small number of substitutions, we also mark those branches with a marginal significance (*p *of 0.05~0.1) by "b".

*PIPSL *shows a remarkably heterogeneous evolutionary pattern when comparing both the *PIP5K1A*-derived and *PSMD4*-derived sequences, and when comparing different species (Figure 2, Table 5). Specifically, the *PIP5K1A*-derived region did not show *Ka*/*Ks *significantly different from 1 for any branch except there is an excess of synonymous mutations in orangutan (Likelihood Ratio Test, LRT *p *= 0.02, Additional file 4). On the other hand, branch-model of CODEML shows heterogeneity of *Ka/Ks *in the *PSMD4*-derived region. A parameter-rich model with internal branches and external branches that has two sets of *Ka/Ks *fits the data better than a one *Ka/Ks *model (*p *= 0.026). Regarding individual species, the *PSMD4*-derived region seems to show negative selection with *Ka*/*Ks *of 0.44 in orangutan although the test is not significant (*p*~0.10). By contrast, it showed a strong signature of adaptive evolution especially prior to the speciation of human and chimp, where 7.1 nonsynonymous substitutions occurred without any synonymous substitutions (*p *= 0.033). If we pool all three ancestral branches leading to human/chimp together, the LRT is still significant with *Ka*/*Ks *of 3.7 and *p *of 0.043, which suggests a long term adaptation of *PSMD4*-derived regions in the ancestors of human and chimp.

**Table 5:** Selection of different lineages based on CODEML.

**Species**	***PSMD4*-derived region**	***PIP5K1A*-derived region**
Human	N	N
Chimp	N	N
Human/Chimp ancestor	A	N
Gorilla	N/A	N
Orangutan	C	C
Gibbon	N	N

N, A and C are short for "failure to reject Neutral null model", "Adaptive" and "Constrained", respectively. Selection force for PSMD4-derived region of gorilla is still unknown given the lack of trace data.

#### (2) Site-specific positive selection

Based on the branch-site model of CODEML, we inferred *PSMD4*-derived region consists of seven nonsynonymous mutations that occurred before the split of human and chimp inferred. Four out of them happened around the second ubiquitin-interaction motif (UIM-2) [21,22], R->H (amino-acid 262 in PSMD4-derived region), A->V (278), Y->C (279) and Q->L (292) (see Additional file 5). Moreover, strongly supports all these substitutions are driven by adaptive evolution. All of them have a Bayes Empirical Bayes (BEB) probability greater than 0.9, which measures whether the *Ka/Ks *is larger than one for this local region. Out of them, Y->C and Q->L even have a BEB value larger than 0.95. These sites are also supported by HyPhy (Additional file 5). The key residue Ala and Ser are unchanged, which ensures that UIM-2 still maintains its ubiquitin-interaction capability to some extent.

By contrast, the parental protein PSMD4 is nearly unchanged across 70~80 million years' mammalian evolution (Figure 2): there is only one nonsynonymous substitution between primate and dog. Inspection of all available vertebrate lineage sequences shows that *PSMD4 *was always under strong purifying selection, except prior to the split of birds and mammalian (see Additional file 6).

### Testis-enriched transcription of *PIPSL *is tightly-regulated by tissue-differential methylation

#### (1) Testing the hypothesis of permissive testis expression

Testis may provide a transciptionally permissive environment [22], which implies transcription leakage tends to occur in testis more frequently than other tissues. From this prospective, pseudogenes could be more likely expressed in testis. Therefore, testis-specific transcription is not necessarily a signature of functionality. In order to test this possibility, we performed genome-wide profiling of all retroposed pseudogenes in human. In brief, we integrated and improved upon previous strategies [4,8,23] to identify retroposed copies (RPCs). Out of 6,750 RPCs, we generated a highly reliable dataset of 729 retropseudogenes and a less stringent dataset of 5,386 retropseudogenes (see Additional file 7).

According to chromosomal coordinates and strands, we cross-referenced this updated retropseudogene dataset with the exon-array based expression data from UCSC genome browser. Herein, UCSC presents the exon-array data as log-ratios with positive values and negative values indicating above-median expression and below-median expression, respectively. We found human testis is not permissive for retropseudogene transcription. As shown in Table 6, no tissues show a remarkable difference in expression of retropseudogenes. Testis expression of retropseudogenes is even slightly lower than many other tissues.

**Table 6:** Exon-array based retropseudogene expression profile across 11 tissues.

**Tissue**	**Expression intensity of Pseudogenes**^A^	**Number of pseudogenes with highest transcription**^B^	**Number of transcribe-able (>=0.2) pseudogenes**^C^
Breast	0.00	42	39
Cerebellum	0.00	58	63
Heart	0.02	74	54
Kidney	0.00	58	64
Liver	0.01	53	43
Muscle	0.01	58	42
Pancreas	0.00	57	56
Prostate	-0.02	24	43
Spleen	0.00	37	31
Testis	0.00	51	38
Thyroid	0.00	39	39

A. Expression value calculated is a log value, which might be smaller than 0 (See Methods). This column shows the median expression of all pseudogenes in the tissue of interest.

B. This column counts the number of pseudogenes which has the highest expression in the tissue of interest no matter how much this highest expression should be.

C. This column is similar to the previous column except the highest expression for a pseudogenes should be above the criterion of 0.2. This value indicates a presence of expression by manually checking the correlation between EST data and exon-array data on UCSC genome browser.

In addition, the exon-array data confirmed the abundant expression of *PIPSL *in testis, consistent with previous results from northern profiling experiments in testis, liver, lung and many other tissues [13]. The abundance of *PIPSL *amounted to 0.71 in testis as revealed by both independent probesets, while *PIPSL *abundance was lower than 0.2 for all other tissues, indicating trace level transcription. For all 548 pseudogenes with exon array data, 38 (7%) are transcribed in testis above the cutoff of 0.2. However, only six out of them (1%) reaches as high abundance as 0.7 in testis. Moreover, all of these six pseudogenes are also transcribed in some other tissues with the abundance above 0.2. Thus, abundant and specific transcription of *PIPSL *establishes it as a clear outlier compared to retropseudogenes.

#### (2) Detecting methylation of *PIPSL*

If testis-specific transcription of *PIPSL *has functional significance, how is this tissue-specificity achieved? Weber and his colleagues generated genome-wide methylation data presented as methylation log2 ratios of bound over input signals and they also proposed the value of 0.4 as a cutoff to differentiate hypermethylation from hypomethylation [24]. As a result, they found germline-specific genes preferentially undergo *de novo *methylation. Specifically, genes that are not methylated in sperm are more likely to get methylated in somatic cells and transcriptionally repressed. Remarkably, *PIPSL *is consistent with this pattern in that it is hypermethylated in primary lung fibroblast cells with the log value of 0.6 and hypomethylated in sperm with the log value of -0.4.

For the aforementioned 729 retropseudogenes, only three of them are covered by Weber *et al*'s data and none of them displays such a *de novo *methylation pattern. Notably, regarding the larger dataset of 5,386 pseudogenes, for which 190 entries are included in Weber *et al*'s set, only ten (5%) show a strong *de novo *methylation in somatic cells as *PIPSL *does. Considering this dataset might include some functional retrogenes, the percentage of real pseudogenes possessing *de novo *methylation might be smaller than 5%. Again, this analysis suggests that *PIPSL *is an intriguing outlier in that *PIPSL*'s transcription profile is highly regulated rather than transcriptionally leaky. That means testis-specific expression could be a functional signature for *PIPSL*.

## Discussion

Babushok *et al*. suggests that the indel around the original start codon disrupts the coding potential of *PIPSL *locus in human. However, our population genetics analysis shows the downstream 2,589 bp ORF is more constrained than the flanking regions in terms of frequency of SNP or indels. Specially, lack of indels in CDS in the current human population not only suggests *PIPSL*'s coding potential, but also indicates that human *PIPSL *is still under purifying selection rather than relaxation, as proposed by Babushok *et al*. Secondly, our forward simulation shows it is highly unlikely that a neutral segment of such a length would remain over 18 million years. As shown by the *PIPSL *locus in gibbon, there are up to three nonsense substitutions and one in-frame indel scatters across the whole region. Thirdly, positive selection always occurred in the internal branches leading to human and chimp, which reveals continuing gain of function for a long time rather than limited to the hominoid ancestor. All these three lines of evidence suggest human *PIPSL *is a bona-fide protein-coding gene. Protein translation might be finely tuned and limited to specific time point, producing a small quantity of protein, as in a special stage of spermatogenesis or during sperm-egg interaction. Thus, *PIPSL *escapes detection of western blotting in whole testis lysates.

The aforementioned comparative analysis also indicates lineage specific evolution after origination of the *PIPSL *locus. Different hominoid species might face different environmental changes, which affect fitness of the same gene, for example, *PIPSL*. An alternative interesting reason might be the difference of the long term effective population size (*N*_*e*_). All branches from the divergence of orangutan and human/chimp/gorilla groups have an estimation of *Ne *which ranges from 10,000 to 100,000 (see Additional file 8) [25-28]. Organisms with a large *N*_*e *_are selectively efficient: those slightly advantageous alleles would be more likely to be fixed and those slightly deleterious mutations would be more likely to be removed [29,30]. By contrast, in organisms with a small *N*_*e*_, slightly beneficial mutations have high chance to get lost and slightly deleterious mutations have high chance to get fixed. From this point of view, *PIPSL *has a small fitness advantage, which is more likely to get fixed or maintained in orangutan or ancestral branches. In contrast, it might be lost in gorilla with a smaller *N*_*e*_. Herein, positive selection might occur before the split of gorilla and human/chimp since they share the majority of their evolutionary history. Thus, two independent losses of the open reading frame occurred in both gorilla and gibbon. It is notable such parallel loss is not that unlikely for new genes. For example, an X-linked testes chimeric gene, *Hun*, was created about 2~3 million years ago, prior to the the split of *D. simulans*, *D. sechellia*, and *D. Mauritiana *[31]. However, *Hun *maintains a integral open reading frame only in *D. simulans*, while its suffers from different frame-distrupting mutations in both *D. sechellia *and *D. Mauritiana*.

As revealed by the lower number of SNPs and indels in human, adaptive selection in the chimp/human ancestral branch (with a much larger *Ne*) might increase the fitness of *PIPSL *to an extent such that it could be maintained in the current population under selective constraint.

Selection drives the remarkable change of UIM-2 on the evolutionary branch leading to human and chimp. Considering UIM-2 while not UIM-1 is the preferred ubiquitin binding partner, maintenance of the essential key residues Ala and Ser in UIM-2 explains why PIPSL still can bind ubiquitin, although its capability does decrease relative to PSMD4 [13]. UIM-2 is also known to be responsible to bind ubiquitin receptors [32]. The remarkable adaptive changes in the ancestor of human and chimp might contribute to the change of interaction partner of PIPSL. Since the parental gene *PSMD4 *undergoes strong purifying selection during hundreds of millions of years of evolution, *PIPSL *could be an interesting target for further comparative functional study.

Finally, it is interesting to ask how many retroposed fused genes the genome encodes considering the prevalence of transcription-mediated gene fusion event [9]. We compared Ensembl gene annotation and retroposed copies we identified, and found *PIPSL *is the unique case in the human genome. Analogously, we do not find any case in rhesus monkey, rat, dog, cow, opossum, platypus and fruitfly. In mouse, we found another case that transcripts of 6030436E02Rik and C330019G07Rik together with 1 kb intergenic region fused first and retroposed to Chromosome 8 (see Additional file 9). The fused locus has been pseudogenized with seven frame shifts and four nonsense mutations or six frame shifts and two nonsense mutations scattered in the 6030436E02Rik-derived region and C330019G07Rik-derived region, respectively. Its non-functionality is also supported by the lack of transcription evidence like EST or mRNA. Extremely low abundance of retroposed, fused genes across numerous animals suggests the inefficiency or complexity of this generation mechanism itself. First, as Akiva *et al*. [9] shows, most transcription-mediated events are rare or confined to certain tissues. In other words, they might not be expressed in the germ line. Second, the mouse case suggests that the fusion might not be able to generate a continuous ORF with intervention of noncoding regions. Akiva also shows only 25% cases can generate a fused ORF. Finally, such locus might not get fixed in the genome considering it interrupts the original dosage balance for multiple genes. In addition to these issues, it is also not clear how retroposed fused copies can insert into an appropriate genomic context to become transciptionally active.

## Conclusion

Thus, *PIPSL *represents an extraordinary case in which natural selection has increased the genetic novelty via a complicated mechanism in primates.

## Methods

Identification of retroposed copies are described in the supplementary methods (see Additional file 7).

### Related bioinformatic databases or resources

We used several annotation tracks of UCSC genome browser like Chip-chip data of Ludwig institute [14] and Human Exon 1.0 ST panel data of Affymetrix [33]. As for the exon array data, UCSC processed the raw signal intensity with a quantile normalization method and generated the summary signal using the PLIER algorithm [34]. After that, these summary values were converted to log-ratios, namely, negative values indicate below-median expression and positive value indicates above-median expression.

### DNA sequencing and population genetics

In order to test functional constraint, we sequenced *PIPSL *in 39 human individuals. DNA samples were purchased from the Coriell Institute for Medical Research, which consists of 10 African Americans, nine Africans in the south of the Sahara, 10 Russians and 10 Chinese. Such a combination should be able to cover the majority of human diversity. *PIPSL *locus including the coding sequence (CDS) and 1 Kb flanking regions (mainly untranslated regions, UTR) were PCR amplified based on primers designed by Oligo . If necessary, multiple PCR experiments were run to amplify the full-length region. After that, PCR bands were sent to Invitrogen for sequencing. For each copy, six to eight walking reactions were performed. Subsequently, we implemented a well-established pipeline including Phred, Phrap [35] and Consed [36] to assemble *PIPSL *locus for each individual.

Single nucleotide polymorphisms (SNPs) and Insertion/Deletions (indels) were identified with Polyphred [37] and Polyscan [38]. Specifically, homozygous or heterozygous SNPs were called by Polyphred first. We retained those highly reliable SNPs with Polyphred score of 99. For SNPs with a score lower than 99, we retained them only if they were identified by Polyscan too. As for indels, we used Polyscan's results because Polyphred failed to identify any indel. After that, we manually checked Polyscan's results and accepted those indels with high scores. Notably, we found Polyscan tends to assign homozygous indels with much higher score by investigating the raw sequencing data. Thus, our strategy tends to overlook heterozygous indels. However, it should not matter that much because our population analysis mainly relies on SNPs rather than indels and such a bias should exist for both coding region and non-coding region.

Finally, we used DnaSP v4.50 [39] to generate the statistics of polymorphisms and perform Hudson, Kreitman and Aguadé's (HKA) test [40] to detect whether both loci follow the neutral null model. In brief, based on number of segregating sites, *S*_*i *_and number of divergences, *d*_*i*_, HKA jointly estimates *θ*_*i*_(selection coefficient), *f*(ratio of *N*_*e *_for both loci) and t (divergence time shared by both loci) by fitting the expected values and variations of *S*_*i *_and *d*_*i*_. Finally, goodness-of-fit is tested with an approximate chi-square test.

We calculated the probability of the number of observed segregation sites (SNP and indels) in CDS on a hypothetical *θ *(*e.g*. the one in 3' UTR) by following the recursive equations [16]:



Where, *l*, *n *and *s *are defined as the length of region of interest, the number of alleles and the number of segregation sites, respectively. *Q*_*n*_(*i*) indicates the probability that *i *mutations occur when there are *n *ancestral lineages, while *P*_*n*_(*s*) indicates the probability that *s *segregating sites in a sample of *n *individuals.

### Evolutionary analysis of *PIPSL*

We slightly modified Tracembler [41] to automatically retrieve homologous reads from NCBI Trace-BLAST website using *PIPSL *sequence as the query, E-value 10^-10 ^as the cutoff, and gorilla, orangutan, and gibbon reads as the database. Subsequently, all reads were submitted to UCSC BLAT [42] server to check whether the best hit of each read is *PIPSL *rather than its parental genes. We retained reads meeting with the following two criteria: the top hit had to match human *PIPSL *locus; the alignment identity of the second top hit was smaller than that of the top hit. Finally, we fed all the retained reads into the aforementioned Phred, Phrap and Consed pipeline and assembled *PIPSL *in gorilla, orangutan, and gibbon.

Baylor University College of Medicine Human Genome Sequencing Center (BCM-HGSC) and Washington University Genome Sequencing Center (WUGSC) sequenced two chromosomes of one wild-born gibbon female. Given the high sequencing coverage for both chromosomes, we identified segregating sites using Polyphred and Polyscan.

We constructed the multiple sequence alignment of *PIPSL *and its parental genes using MUSCLE [43] and further manually checked the alignment in GeneDoc [44]. Then this protein based alignment was converted to the codon-based alignment with PAL2NAL [45].

We performed evolutionary simulation using ReEVOLVER v1.0 as its online document describes [18]. It estimates the probability that an ORF is maintained for millions of years of evolution. In simulation, we used the species tree described in [18], the substitution rate of 1.0 × 10^-9 ^per site per year and the indel rate of 1.0 × 10^-10 ^per site per year [18]. Given these parameters, ReEVOLVER assumed Kimura-2-parameters model of sequence evolution and did forward simulations from the ancestral sequence constructed by PAML [20] or DNApars [46]. In this process, the so-called disable features, i.e., mutations causing stop codons or frame shifts, are counted. We performed 100,000 simulations for the whole *PIPSL *locus, *PSMD4*-derived region and *PIP5K1A*-derived region, separately.

ML-based analysis were implemented using CODEML of PAML package v4.0b [20]. We used the free-ratio model to estimate number of synonymous site and nonsynonymous sites and branch-model to estimate whether there is a significant departure compared to the neutral expectation along one specific lineage. In order to infer which site is under adaptive evolution in a specific lineage, we re-ran CODEML with the branch-site model. Specifically, Yang and Nielsen implemented two models, called A and B [47], which permits variation of the ω ratio (*i.e*., *Ka/Ks*) both among sites and among lineages. PAML 4.0b further permits ω of the null model to vary between 0 and 1 rather than the old model A which fixes the ω ratio to 0. There are two tests associated with current model A. We used Test 2 (fix_omega = 1; omega = 1), which is supposed to be more robust to differentiate positive selection compared to a relaxation of functional constraint [48]. However, in order to increase confidence, we used SubtreeSelection module of HyPhy [49] to do a similar analysis. Across ReEVOLVER, PAML and HyPhy, we used the species tree of primates described in [18].

As a complementary test, we also estimated the time required to destroy *PIPSL*'s ORF in one half of all simulations, *t*_1/2 _[19]. We followed the same substitution rate or indel rate as [19] and found *t*_1/2 _is about 1.9 million years. Thus, considering *PIPSL *predated diverge of human and gibbon 18 million years ago, the possibility to maintain this ORF is like 0.5^18/1.9 ^or 1 × 10^-3^.

## Authors' contributions

YZ performed all analysis. SL did DNA sequencing. SZ involved in identification of retroposed copies. YZ, XZ, ML and LW designed and drafted the manuscript. All authors read and approved the final manuscript.

## Supplementary Material

Additional file 1 **Text-format of polymorphism distribution**. Polymorphism including SNPs and indels across 39 individuals. Click here for file

Additional file 2 **Snapshot of *PIPSL *locus with Chip-chip tracks from UCSC genome browser**. Four tracks are attached, which show the validated results of Ludwig Institute Chip-chip experiments. Small blocks show signal of biding sites with darker color indicating stronger binding affinity. Light lavender block, light blue block and light yellow block mark the sequenced 5' promoter, 5' UTR and 3' UTR, respectively. Click here for file

Additional file 3 **Alignment of the complete *PIPSL *locus across all five all hominoids and the parental genes from human, chimp and dog**. The number on top and left indicate the location of nucleotides in alignment and member sequence, respectively. "ATG" and "TGA" in yellow mark the start and stop codon. Nucleotides in red mark the codons in gibbon and gorilla with some disrupting mutations. Click here for file

Additional file 4 **Parameters for likelihood ratio tests in CODEML**. Parameters we used in CODEML are summarized. Click here for file

Additional file 5 **Alignment of *PSMD4*-derived region and sites under positive selection detected by HyPhy**. For panel A, we showed the alignment of PSMD4-derived region. Yellow bars and red bars marks the mutations occurred in the ancestor of all hominoids and those occurred before the split of human and chimp, respectively. Light blue rectangle define the core residues of UIM-1 (LALALS) and UIM-2 (IAYAMS). The numbers above red bars show the probability calculated by BEB statistics with "*" as a label of significance. For panel B, We used the "SubtreeSelectionComparason" module of HyPhy to infer the sites driven by positive selection on the internal branch, the split of human/chimp and orangutan. The blue and red lines indicate approximate site-specific dN and corresponding Log10-based LRT *p*, respectively. The yellow line marks the confidence level of p of 0.05. Compared to CODEML, HyPhy identified more sites under adaptive selection. As a remarkable overlap between both methods, UIM-2 is remarkably enriched with such sites. Click here for file

Additional file 6 **Evolution of *PSMD4 *across various vertebrates**. The pink rectangle marks the PIPSL locus, while the blue bar marks the adaptive evolution of PSMD4 before the split of birds and mammals. The number on all the branches like (#0.0090) shows the *Ka/Ks*. Click here for file

Additional file 7 **Supplementary methods**. We described the methodology in identification of retroposed copies and expressional profiling. Click here for file

Additional file 8 **Phylogeny of hominoids with estimation of effective population size marked**. Numbers in red marks the divergence time in million years, while numbers in black show the estimation of *N*_*e*_. Click here for file

Additional file 9 **A similar retroposed fused locus in mouse**. 6030436E02Rik and C330019G07Rik encoded by chromosome 5 were co-retroposed to chromosome 8. Click here for file

## References

[B1] Long M, Betran E, Thornton K, Wang W (2003). The origin of new genes: glimpses from the young and old. Nat Rev Genet.

[B2] Li W-H (1997). Molecular Evolution.

[B3] Betran E, Thornton K, Long M (2002). Retroposed new genes out of the X in Drosophila. Genome Res.

[B4] Marques AC, Dupanloup I, Vinckenbosch N, Reymond A, Kaessmann H (2005). Emergence of young human genes after a burst of retroposition in primates. PLoS Biol.

[B5] Dai H, Chen Y, Chen S, Mao Q, Kennedy D, Landback P, Eyre-Walker A, Du W, Long M (2008). The evolution of courtship behaviors through the origination of a new gene in Drosophila. Proceedings of the National Academy of Sciences.

[B6] Bai Y, Casola C, Feschotte C, Betran E (2007). Comparative genomics reveals a constant rate of origination and convergent acquisition of functional retrogenes in Drosophila. Genome Biol.

[B7] Zhang J, Dean AM, Brunet F, Long M (2004). Evolving protein functional diversity in new genes of Drosophila. Proc Natl Acad Sci USA.

[B8] Vinckenbosch N, Dupanloup I, Kaessmann H (2006). Evolutionary fate of retroposed gene copies in the human genome. Proceedings of the National Academy of Sciences.

[B9] Akiva P, Toporik A, Edelheit S, Peretz Y, Diber A, Shemesh R, Novik A, Sorek R (2006). Transcription-mediated gene fusion in the human genome. Genome Res.

[B10] Loijens JC, Anderson RA (1996). Type I phosphatidylinositol-4-phosphate 5-Kinases are distinct members of this Novel Lipid Kinase Family. J Biol Chem.

[B11] Heck JN, Mellman DL, Ling K, Sun Y, Wagoner MP, Schill NJ, Anderson RA (2007). A conspicuous connection: structure defines function for the phosphatidylinositol-phosphate kinase family. Crit Rev Biochem Mol Biol.

[B12] Wang Q, Young P, Walters KJ (2005). Structure of S5a bound to monoubiquitin provides a model for polyubiquitin recognition. J Mol Biol.

[B13] Babushok DV, Ohshima K, Ostertag EM, Chen X, Wang Y, Mandal PK, Okada N, Abrams CS, Kazazian HH (2007). A novel testis ubiquitin-binding protein gene arose by exon shuffling in hominoids. Genome Res.

[B14] Kim TH, Barrera LO, Zheng M, Qu C, Singer MA, Richmond TA, Wu Y, Green RD, Ren B (2005). A high-resolution map of active promoters in the human genome. Nature.

[B15] Hartl DL, Clark AG (2007). Human population genetics. Principles of population genetics.

[B16] Hudson RR (1990). Gene genealogies and the coalescent process. Oxford Surveys in Evolutionary Biology 7 Oxford.

[B17] Gillespie JH (2004). Population Genetics: A Concise Guide.

[B18] Dupanloup I, Kaessmann H (2006). Evolutionary simulations to detect functional lineage-specific genes. Bioinformatics.

[B19] Zhang J, Webb DM (2003). Evolutionary deterioration of the vomeronasal pheromone transduction pathway in catarrhine primates. Proceedings of the National Academy of Sciences.

[B20] Yang Z (2007). PAML 4: phylogenetic analysis by maximum likelihood. Mol Biol Evol.

[B21] Hawryluk MJ, Keyel PA, Mishra SK, Watkins SC, Heuser JE, Traub LM (2006). Epsin 1 is a polyubiquitin-selective clathrin-associated sorting protein. Traffic.

[B22] Schmidt EE (1996). Transcriptional promiscuity in testes. Curr Biol.

[B23] Zhang Z, Carriero N, Zheng D, Karro J, Harrison PM, Gerstein M (2006). PseudoPipe: an automated pseudogene identification pipeline. Bioinformatics.

[B24] Weber M, Hellmann I, Stadler MB, Ramos L, Paabo S, Rebhan M, Schubeler D (2007). Distribution, silencing potential and evolutionary impact of promoter DNA methylation in the human genome. Nature Genetics.

[B25] Yu N, Jensen-Seaman MI, Chemnick L, Ryder O, Li WH (2004). Nucleotide diversity in Gorillas. Genetics.

[B26] Chen FC, Li WH (2001). Genomic divergences between humans and other hominoids and the effective population size of the common ancestor of humans and chimpanzees. Am J Hum Genet.

[B27] Hobolth A, Christensen OF, Mailund T, Schierup MH (2007). Genomic relationships and speciation times of human, chimpanzee, and gorilla inferred from a coalescent hidden Markov model. PLoS Genet.

[B28] Fischer A, Pollack J, Thalmann O, Nickel B, Paabo S (2006). Demographic history and genetic differentiation in apes. Curr Biol.

[B29] Kimura M (1985). Neutral Theory of Molecular Evolution.

[B30] Lynch M (2007). The Origins of Genome Architecture.

[B31] Arguello JR, Chen Y, Yang S, Wang W, Long M (2006). Origination of an X-linked testes chimeric gene by illegitimate recombination in Drosophila. PLoS Genet.

[B32] Kang Y, Chen X, Lary JW, Cole JL, Walters KJ (2007). Defining how ubiquitin receptors hHR23a and S5a bind polyubiquitin. J Mol Biol.

[B33] Human Exon Array 1.0 ST Panel. http://www.affymetrix.com/products_services/arrays/specific/exon.affx.

[B34] Guide to Probe Logarithmic Intensity Error (PLIER) Estimation. http://www.affymetrix.com/support/technical/technotes/plier_technote.pdf.

[B35] Ewing B, Green P (1998). Base-calling of automated sequencer traces using phred. II. Error probabilities. Genome Res.

[B36] Gordon D, Abajian C, Green P (1998). Consed: a graphical tool for sequence finishing. Genome Res.

[B37] Nickerson DA, Tobe VO, Taylor SL (1997). PolyPhred: automating the detection and genotyping of single nucleotide substitutions using fluorescence-based resequencing. Nucleic Acids Res.

[B38] Chen K, McLellan MD, Ding L, Wendl MC, Kasai Y, Wilson RK, Mardis ER (2007). PolyScan: an automatic indel and SNP detection approach to the analysis of human resequencing data. Genome Res.

[B39] Rozas J, Sanchez-DelBarrio JC, Messeguer X, Rozas R (2003). DnaSP, DNA polymorphism analyses by the coalescent and other methods. Bioinformatics.

[B40] Hudson RR, Kreitman M, Aguade M (1987). A test of neutral molecular evolution based on nucleotide data. Genetics.

[B41] Dong Q, Wilkerson MD, Brendel V (2007). Tracembler-software for in-silico chromosome walking in unassembled genomes. BMC Bioinformatics.

[B42] Kent WJ (2002). BLAT--the BLAST-like alignment tool. Genome Res.

[B43] Edgar RC (2004). MUSCLE: multiple sequence alignment with high accuracy and high throughput. Nucleic Acids Res.

[B44] Nicholas KB, Nicholas HB, Deerfield DW (1997). GeneDoc: analysis and visualization of genetic variation. EMBNEW NEWS.

[B45] Suyama M, Torrents D, Bork P (2006). PAL2NAL: robust conversion of protein sequence alignments into the corresponding codon alignments. Nucleic Acids Res.

[B46] Retief JD (2000). Phylogenetic analysis using PHYLIP. Methods Mol Biol.

[B47] Yang Z, Nielsen R (2002). Codon-substitution models for detecting molecular adaptation at individual sites along specific lineages. Mol Biol Evol.

[B48] Zhang J, Nielsen R, Yang Z (2005). Evaluation of an Improved Branch-Site Likelihood Method for Detecting Positive Selection at the Molecular Level. Mol Biol Evol.

[B49] Pond SL, Frost SD, Muse SV (2005). HyPhy: hypothesis testing using phylogenies. Bioinformatics.

