# Supplementary material for: Positive selection for the male functionality of a co-retroposed gene in the hominoids
Source: BMC Evol Biol. 2009 Oct 15;9:252. doi: 10.1186/1471-2148-9-252 (PMC2773790; doi:10.1186/1471-2148-9-252)
Supplement: Additional file 2 — Snapshot of PIPSL locus with Chip-chip tracks from UCSC genome browser. Four tracks are attached, which show the validated results of Ludwig Institute Chip-chip experiments. Small blocks show signal of biding sites with darker color indicating stronger binding affinity. Light lavender block, light blue block and light yellow block mark the sequenced 5' promoter, 5' UTR and 3' UTR, respectively. [file 1471-2148-9-252-S2.pdf]

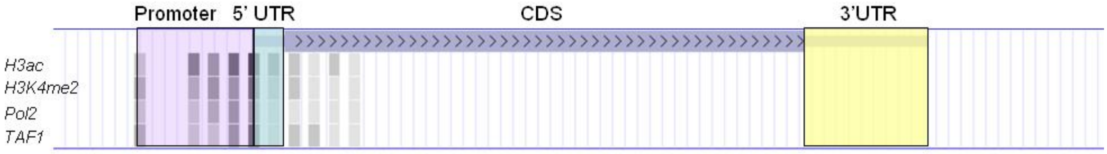

Snapshot of *PIPSL* locus from UCSC genome browser [1, 2]. Four tracks are attached, which show the validated results of Ludwig Institute Chip-chip experiments. Small blocks show signal of biding sites with darker color indicating stronger binding affinity. Light lavender block, light blue block and light yellow block mark the sequenced 5' promoter, 5' UTR and 3' UTR, respectively.

1. Kuhn RM, Karolchik D, Zweig AS, Trumbower H, Thomas DJ, Thakkapallayil A, Sugnet CW, Stanke M, Smith KE, Siepel A *et al*: **The UCSC genome browser database: update 2007**. *Nucleic Acids Res* 2007, **35**(Database issue):D668-673.
2. Kim TH, Barrera LO, Zheng M, Qu C, Singer MA, Richmond TA, Wu Y, Green RD, Ren B: **A high-resolution map of active promoters in the human genome**. *Nature* 2005, **436**(7052):876.
