# Supplementary material for: Positive selection for the male functionality of a co-retroposed gene in the hominoids
Source: BMC Evol Biol. 2009 Oct 15;9:252. doi: 10.1186/1471-2148-9-252 (PMC2773790; doi:10.1186/1471-2148-9-252)
Supplement: Additional file 3 — Alignment of the complete PIPSL locus across all five all hominoids and the parental genes from human, chimp and dog. The number on top and left indicate the location of nucleotides in alignment and member sequence, respectively. "ATG" and "TGA" in yellow mark the start and stop codon. Nucleotides in red mark the codons in gibbon and gorilla with some disrupting mutations. [file 1471-2148-9-252-S3.pdf]

```
             1160        *       1180        *       1200        *       1220        *       1240        *       1260
gibbon.fas : ATGGCATCTGAGGTGCCTTATGCCTCTGGCATGCCCATCAAGAAAACAGGCCATAGAAGTGTTGATTCCTCAGGAGAGACAACATATAAAAAGACAACCTCATCA :  907
pongo.fast : ATGGCATCTGAGGTGCCTTATGCCTCTGGCATGCCCATCAAGAAAATAGGCCATAGAAGTGTTGATTCCTCAGGAG---------------AGACAACCTCATCA : 1074
gorilla.fa : ATGGCATCTGAGGTGCCTTATGCCTCTGGCATGCCCATCAAGAAAATAGGCCATAGAAGTGTTGATTCCTCAGGAG---------------AGACAACCTCATCA :  410
chimp.fast : ATGGCATCTGAGGTGCCTTATGCCTCTGGCATGCCCATCAAGAAAATAGGCCATAGAAGTGTTGATTCCTCAGGAG---------------GGACAACCTCATCA : 1210
hg18_dna   : ATGGCATCTGAGGTGCCTTATGCCTCTGGCATGCCCATCAAGAAAATAGGCCATAGAAGTGTTGATTCCTCAGGAG---------------GGACAACCTCATCA : 1234
human_pare : ATGGCATCTGAGGTGCCTTATGCCTCTGGCATGCCCATCAAGAAAATAGGCCATAGAAGTGTTGATTCCTCAGGAGAGACAACATATAAAAAGACAACCTCATCA :  210
chimp_pare : ATGGCATCTGAGGTGCCTTATGCCTCTGGCATGCCCATCAAGAAAATAGGCCATAGAAGTGTTGATTCCTCAGGAG---------------AGACAACCTCATCA :  198
dog_parent : GTGGGGTCTGAGGTGCCTTATGCCTCTGGCATGCCCATCAAGAAAATAGGCCACCGAGGTGTTGATTCCTCAGGAGAGACAACATATAAAAAGACAACCTCATCT :  207
             aTGGcaTCTGAGGTGCCTTATGCCTCTGGCATGCCCATCAAGAAAAtAGGCCAtaGAaGTGTTGATTCCTCAGGAG               aGACAACCTCATCa

                     *       1280        *       1300        *       1320        *       1340        *       1360
gibbon.fas : GCCTTGAAAGGTGCCATCCAGTTAGGCATTACCCACACTGTGGGGAGCCTGAGTACCAAACCAGAGAGTGATGTCCTCATGCAAGATTTCCACATGGTGGAGAGT : 1012
pongo.fast : GCCTTGAAAGGTGCCATCCAGTTAGGCATTACCCACACTGTGGGGAGCCTGAGTACCAGACCAGAGAGTGATGTCCTCATGCAAGATTTCCACATGGTGGAGAGT : 1179
gorilla.fa : GCCTTGAAAGGTGCCATCCAGTTAGGCATTACCCACACTGTGGGGAGCCTGAGTACCAAACCAGAGAGTGATGTCCTCATGCAAGATTTCCACATGGTGGAGAGT :  515
chimp.fast : GCCTTGAAAGGTGCCATCCAGTTAGGCATTACCCACACTGTGGGGAGCCTGAGTACCAAACCAGAGAGTGATGTCCTCATGCAAGATTTCCACATGGTGGAGAGT : 1315
hg18_dna   : GCCTTGAAAGGTGCCATCCAGTTAGGCATTACCCACACTGTGGGGAGCCTGAGTACCAAACCAGAGAGTGATGTCCTCATGCAAGATTTCCACATGGTGGAGAGT : 1339
human_pare : GCCTTGAAAGGTGCCATCCAGTTAGGCATTACCCACACTGTGGGGAGCCTGAGTACCAAACCAGAGCGTGATGTCCTCATGCAAGATTTCTACGTGGTTGAGAGT :  315
chimp_pare : GCCTTGAAAGGTGCCATCCAGTTAGGCATTACCCACACTGTGGGGAGCCTGAGTACCAAACCAGAGCGTGATGTCCTCATGCAAGATTTCTACGTGGTTGAGAGT :  303
dog_parent : GCCTTGAAAGGTGCCATCCAGTTAGGCATTACTCACACTGTGGGAAGCCTGAGTACCAAACCAGAGCGTGATGTCCTCATGCAAGACTTCTATGTGGTGGAGAGT :  312
             GCCTTGAAAGGTGCCATCCAGTTAGGCATTACcCACACTGTGGGgAGCCTGAGTACCAaACCAGAG GTGATGTCCTCATGCAAGAtTTC Ac TGGTgGAGAGT

                 *       1380        *       1400        *       1420        *       1440        *       1460        *
gibbon.fas : ATCTTCTTTCCCAGTGAAGGGAGCAACCTGACCCCTGCTCATCACTACAATGCCTTTAGTTTCAAGACCTATGCACCTGTTGCCTTCCGCTACTTCCGGGAGCTA : 1117
pongo.fast : ATCTTCTTTCCCAGTGAAGGGAGCAACCTGACCCCTGCTCATCACTACAATGACTTTCGTTTCAAGACCTATGCACCTGTTGCCTTCCGCTACTTCTGGGAGCTA : 1284
gorilla.fa : ACCTTCTTTCCCAGTGAAGGGAGCAACCTGACCCCTGCTCATCACTACAATGACTTTCGTTTCAAGACCTATGCACCTGTTGCCTTCCGCTACTTCTGGAAGCTA :  620
chimp.fast : ATCTTCTTTCCCAGTGAAGGGAGCAACCTGACCCCTGCTCATCACTACAATGCCTTTCGTTTCAAGACCTATGCACCTGTTGCCTTCCGCTACTTCTGGGAGCTA : 1420
hg18_dna   : ATCTTCTTTCCCAGTGAAGGGAGCAACCTGACCCCTGCTCATCACTACAATGCCTTTCGTTTCAAGACCTATGCACCTGTTGCCTTCCGCTACTTCTGGGAGCTA : 1444
human_pare : ATCTTCTTTCCCAGTGAAGGGAGCAACCTGACCCCTGCTCATCACTACAATGACTTTCGTTTCAAGACCTATGCACCTGTTGCCTTCCGCTACTTCCGGGAGCTA :  420
chimp_pare : ATCTTCTTTCCCAGCGAAGGGAGCAACCTGACCCCTGCTCATCACTACAATGACTTTCGTTTCAAGACCTATGCACCTGTTGCCTTCCGCTACTTCCGGGAGCTA :  408
dog_parent : ATCTTCTTCCCCAGTGAAGGGAGTAACTTGACCCCTGCTCATCACTACAATGACTTTCGGTTCAAGACCTATGCACCTGTTGCCTTCCGTTACTTTCGGGAGTTA :  417
             AtCTTCTTtCCCAGtGAAGGGAGcAACcTGACCCCTGCTCATCACTACAATG CTTTcGtTTCAAGACCTATGCACCTGTTGCCTTCCGcTACTTc GGgAGcTA

                    1480        *       1500        *       1520        *       1540        *       1560        *
gibbon.fas : TTTGGTATCCGGCCCGATGACTACTTATATTCCCTCTGCAGTGAGCCGCTGATTGAACTCTGTAGCTCTGGAGCTAGTGGTTCCCTGTTCTATGTGTCCAGCGAC : 1222
pongo.fast : TTTGGTATCCGGCCCGATGATTACTTGTATTCCCTCTGCAGTGAGCCGCTGATTGAACTCTGTAGCTCTGGAGCTAGTGGTTCCCTGTTCTATGCGTCCGGCGAC : 1389
gorilla.fa : TTTGGTATCCGGCCCGATGATTACTTGTATTCCCTCTGCAGTGAGCCGCTGATTGAACTCTGTAGCTCTGGAGCTAGTGGTTCCCTGTTCTATGTGTCCAGCGAC :  725
chimp.fast : TTTGGTATCCGGCCCAGTGATTACTTGTATTCCCTCTGCAGTGAGCCGCTGATTGAACTCTGTAGCTCTGGAGCTAGTGGTTCCCTGTTCTATGTGTCCAGCGAC : 1525
hg18_dna   : TTTGGTATCCGGCCCGATGATTACTTGTATTCCCTCTGCAGTGAGCCGCTGATTGAACTCTGTAGCTCTGGAGCTAGTGGTTCCCTGTTCTATGTGTCCAGCGAC : 1549
human_pare : TTTGGTATCCGGCCCGATGATTACTTGTATTCCCTCTGCAGTGAGCCGCTGATTGAACTCTGTAGCTCTGGAGCTAGTGGTTCCCTATTCTATGTGTCCAGCGAC :  525
chimp_pare : TTTGGTATCCGGCCCGATGATTACTTGTATTCCCTCTGCAGTGAGCCGCTGATTGAACTCTGTAGCTCTGGAGCTAGTGGTTCCCTATTCTATGTGTCCAGCGAC :  513
dog_parent : TTTGGTATCCGGCCTGATGATTACTTGTACTCCCTTTGCAATGAACCACTGATTGAACTCTGCAGCTCTGGGGCTAGTGGTTCCCTCTTCTACGTGTCCAGTGAC :  522
             TTTGGTATCCGGCCcgaTGAtTACTTgTAtTCCCTcTGCAgTGAgCCgCTGATTGAACTCTGtAGCTCTGGaGCTAGTGGTTCCCT TTCTAtGtGTCCaGcGAC

             1580        *       1600        *       1620        *       1640        *       1660        *       1680
gibbon.fas : GATGAGTTCATTATTAAGACAGTCCAACATAAAGAGGCGGAGTTTCTGCAGAAGCTGCTTCCAGGATACTACATGAACTTCAACCAGAACCCTCGGACTTTGCTG : 1327
pongo.fast : GATGAGTTCATTATTAAGACAGTCCGACATAAAGAGGCAGAGTTTCTGCAGAAGCTGCTTCCAGGATACTACATGAACCTCAACCAAAACCCTCGGACTTTGCTG : 1494
gorilla.fa : GATGAGTTCATTATTAAGACAGTCTGACATAAAGAGGCGGAGTTTCTGCAGAAGCTGCTTCCAGGATACTACATAAACCTCAACCAGAACCCTCGGACTTTGCTG :  830
chimp.fast : GATGAGTTCATTATTAAGACAGTCCGACATAAAGAGGCGGAGTTTCTGCAGAAGCTGCTTCCAGGATACTACATAAACCTCAACCAGAACCCTCGGACTTTGCTG : 1630
hg18_dna   : GATGAGTTCATTGTTAAGACAGTCCGACATAAAGAAGCGGAGTTTCTGCAGAAGCTGCTTCCAGGATACTACATAAACCTCAACCAGAACCCTCGGACTTTGCTG : 1654
human_pare : GATGAGTTCATTATTAAGACAGTCCAACATAAAGAGGCGGAATTTCTGCAGAAGCTGCTTCCAGGATACTACATGAACCTCAACCAGAACCCTCGGACTTTGCTG :  630
chimp_pare : GATGAGTTCATTATTAAGACAGTCCAACATAAAGAGGCGGAATTTCTGCAGAAGCTGCTTCCAGGATACTACATGAACCTCAACCAGAACCCTCGGACTTTGCTG :  618
dog_parent : GATGAATTCATCATCAAAACGGTCCAGCATAAAGAGGCGGAGTTTCTGCAGAAGCTGCTTCCAGGATACTACATGAACCTCAACCAGAATCCTCGCACTTTGCTA :  627
             GATGAgTTCATtaTtAAgACaGTCc aCATAAAGAgGCgGAgTTTCTGCAGAAGCTGCTTCCAGGATACTACAT AACcTCAACCAgAAcCCTCGgACTTTGCTg

                     *       1700        *       1720        *       1740        *       1760        *       1780
gibbon.fas : CCTAAATTTTATGGACTGTACTGTGTGCAGGTAGGTGACAAGAACATTCGGATTGTGGTGATGAACAATCTTTTACCAAGATCGGTAAAAATGCATATCAAATAT : 1432
pongo.fast : CCTAAATTCTATGGACTGTACTGTGTGCAGACAGGTGGCAAGAACATTCGGATTGTGGTGATGAACAATCTTTTACCAAGGTCGGTAAAAATGCATATCAAATAT : 1599
gorilla.fa : CCTAAATTCTATGGACTGTACTGTGTGCAGGCAGGTGGCAAGAACATTCGGATTGTGGTGATGAACAATCTTTCACCAAGATCGGTAAAAATGCATATCAAATAT :  935
chimp.fast : CCTAAATTCTATGGACTGTACTGTGTGCAGGCAGGTGGCAAGAACATTCGGATTGTGGTGATGAACAATCTTTTACCAAGATCGGTAAAAATGCATATCAAATAT : 1735
hg18_dna   : CCTAAATTCTATGGACTGTACTGTGTGCAGACAGGTGGCAAGAACATTCGGATTGTGGTGATGAACAATCTTTTACCAAGATCGGTAAAAATGCATATCAAATAT : 1759
human_pare : CCTAAATTCTATGGACTGTACTGTGTGCAGGCAGGTGGCAAGAACATTCGGATTGTGGTGATGAACAATCTTTTACCAAGATCGGTAAAAATGCATATCAAATAT :  735
chimp_pare : CCTAAATTCTATGGACTGTACTGTGTGCAGGCAGGTGGCAAGAACATTCGGATTGTGGTGATGAACAATCTTTTACCAAGATCGGTAAAAATGCATATCAAATAT :  723
dog_parent : CCTAAGTTCTATGGACTGTATTGTGTGCAGGCAGGTGGTAAGAACATTCGAATTGTGGTGATGAACAATCTCTTACCACGGTCGGTCAAGATGCACATCAAATAT :  732
             CCTAAaTTcTATGGACTGTAcTGTGTGCAGgcAGGTGgcAAGAACATTCGgATTGTGGTGATGAACAATCTtTtACCAaGaTCGGTaAAaATGCAtATCAAATAT

                 *       1800        *       1820        *       1840        *       1860        *       1880        *
gibbon.fas : GACCTCAAAGGCTCAACCTACAAACGGCGGGCTTCCCAGAAAGAGCGAGAGAAGCCTCTTCCCACATTTAAAGACCTAGACTTCTTACAAGACATCCCTGATGGT : 1537
pongo.fast : GACCTCAAAGGCTCAACCTACAAACGGCGGGCTTCCCAGAAAGAGCGAGAGAAGCCTCTTCCCACATTTAAAGACCTAGACTTCTTACAAGACATCCCTGATGGT : 1704
gorilla.fa : GACCTCAAAGGCTCAACGTACAAACGGCGGGCTTCCCAGAAAGAGCGAGAGAAGCCTCTTCCCACATTTAAAGACCTAGACTTCTTACAAGACATCCCTGATGGT : 1040
chimp.fast : GACCTCAAAGGCTCAACCTACAGACGGCGGGCTTCCCAGAAAGAGCGAGAGAAGCCTCTTCCCACATTTAAAGACCTAGACTTCTTACAAGACATCCCTGATGGT : 1840
hg18_dna   : GACCTCAAAGGCTCAACCTACAGGCGGCGGGCTTCCCAGAAAGAGCGAGAGAAGCCTCTTCCCACATTTAAAGACCTAGACTTCTTACAAGACATCCCTGATGGT : 1864
human_pare : GACCTCAAAGGCTCAACCTACAAACGGCGGGCTTCCCAGAAAGAGCGAGAGAAGCCTCTTCCCACATTTAAAGACCTAGACTTCTTACAAGACATCCCTGATGGT :  840
chimp_pare : GACCTCAAAGGCTCAACCTACAAACGGCGGGCTTCCCAGAAAGAGCGAGAGAAGCCTCTTCCCACATTTAAAGACCTAGACTTCTTACAAGACATCCCTGATGGT :  828
dog_parent : GACCTCAAGGGCTCAACCTACAAACGACGGGCTTCCCAAAAAGAGCGAGAGAAGCCTCTCCCCTACCTTTAAAGACCTAGACTTCTTACAAGACATTCCAGATGGT :  837
             GACCTCAAaGGCTCAACcTACAaaCGgCGGGCTTCCCAgAAAGAGCGAGAGAAGCCTCTtCCcACaTTTAAAGACCTAGACTTCTTACAAGACATcCCtGATGGT

                    1900        *       1920        *       1940        *       1960        *       1980        *
gibbon.fas : CTTTTTTTGGATGCTGACATGCACAATGCTCTCTGTAAGACCCTGCAGCGTGACTGTTTGGTGCTGCAGAGCTTCAAGATAATGGATTATAGCCTCTTGATGTCA : 1642
pongo.fast : CTTTTTTTGGATGCTGACGTGCACAACGCTCTCTGTAAGACCCTGCAGCGTGACTGTTTGGTGCTGCAGAGCTTCAAGATAATGGGTTATAGCCTCTTGATGTCA : 1809
gorilla.fa : CTTTTTTTGGATGCTGACGTGCACAACGCTCTCTGTAAGACCCTGCAGCGTGACTGTTTGGTGCTGCAGAGCTTCAAGATAATGGATTATAGCCTCTTGATGTCA : 1145
chimp.fast : CTTTTTTTGGATGCTGACGTGCACAACGCTCTCTGTAAGACCCTGCAGCGTGACTGTTTGGTGCTGCAGAGCTTCAAGATAATGGATTATAGCCTCTTGATGTCA : 1945
hg18_dna   : CTTTTTTTGGATGCTGACGTGCACAACGCTCTCTGTAAGACCCTGCAGCGTGACTGTTTGGTGCTGCAGAGCTTCAAGATAATGGATTATAGCCTCTTGATGTCA : 1969
human_pare : CTTTTTTTGGATGCTGACATGTACAACGCTCTCTGTAAGACCCTGCAGCGTGACTGTTTGGTGCTGCAGAGCTTCAAGATAATGGATTACAGCCTCTTGATGTCA :  945
chimp_pare : CTTTTTTTGGATGCTGACATGTACAACGCTCTCTGTAAGACCCTGCAGCGTGACTGTTTGGTGCTGCAGAGCTTCAAGATAATGGATTATAGCCTCTTGATGTCA :  933
dog_parent : CTTTTTTTGGATGCTGACATGTACAATGCTCTGTGTAAGACCCTACAGCGTGACTGTTTGGTGTTGCAGAGCTTCAAGATAATGGACTATAGCCTCTTGATGTCA :  942
             CTTTTTTTGGATGCTGAC TG ACAAcGCTCTcTGTAAGACCCTgCAGCGTGACTGTTTGGTGcTGCAGAGCTTCAAGATAATGGatTAtAGCCTCTTGATGTCA

             2000        *       2020        *       2040        *       2060        *       2080        *       2100
gibbon.fas : ATCCATAATATAGATCATGCACAACGAGAGCCCTTAAGCAGCAAAACACAATACTCAGTTGATACTCGAAGACCAGCCCCCCAAAAGGCTCTGTATTCCACAGCC : 1747
pongo.fast : ATCCATAATATAGATCATGCACAACGAGAGCCCTTAAGCAGCGAAACACAATACTCAGTTGATACTGGAAGACCGGCCCCCCAAAAGGCTCTGTATTCCACAGCC : 1914
gorilla.fa : ATCCATAATATAGATCAGGCACAACGAGAGCCCTTAAGCAGCGAAACACAATACTCAGTTGATACTCGAAGACCAGCCCCCCAAAAGGCTCTGTATTCCACAGCC : 1250
chimp.fast : ATCCATAATATAGATCATGCACAACGAGAGCCCTTAAGCAGCGAAACACAATACTCAGTTGATACTCAAAGACCGGCCCCCCAAAAGGCTCCGTATTCCACAGCC : 2050
hg18_dna   : ATCCATAATATAGATCATGCACAACGAGAGCCCTTAAGCAGCGAAACACAATACTCAGTTGATACTCGAAGACCAGCCCCCCAAAAGGCTCTGTATTCCACAGCC : 2074
human_pare : ATCCATAATATAGATCATGCACAACGAGAGCCCTTAAGCAGTGAAACACAGTACTCAGTTGATACTCGAAGACCGGCCCCCCAAAAGGCTCTGTATTCCACAGCC : 1050
chimp_pare : ATCCATAATATAGATCATGCACAACGAGAGCCCTTAAGCAGTGAAACACAGTACTCAGTTGATACTCGAAGACCGGCCCCCCAAAAGGCTCTGTATTCCACAGCC : 1038
dog_parent : ATTCATAACATAGATCACGCACAGCGAGAGCCCTTAGGCAATGAAGCACAGTGCTCAGCTGACACTCGCAAACCAGCCCCCCAAAAGGCCCTCTATTCCACAGCT : 1047
             ATcCATAAtATAGATCAtGCACAaCGAGAGCCCTTAaGCAg gAAaCACA TaCTCAGtTGAtACTcgaAgACC GCCCCCCAAAAGGCtCtgTATTCCACAGCc
```

```
                      *      2120         *      2140         *      2160         *      2180         *      2200
gibbon.fas  : ATGGAATCCATCCAGGGAGAGGCTCGACGGGGTGGCACTGTGGAGACCGATGACCACATGGGTGGCATCCCTGCCCAGAATAGTAAAGGGGAAAGGCTTCTGCTT : 1852
pongo.fast  : ATGGAATCCATCCAGGGAGAGGCTCGAAGGGGTGGCACCATGGAGACCGATGACCATATGGGTGGCATCCCTGCCCGGAATAGTAAAGGGGAAAGGCTTCTGCTT : 2019
gorilla.fa  : ATGGAATCCATCCAGGGAGAGGCTCGACGGGGTGGCACCATGGAGACCGATGACCATATGGGTGGCATCCCTGCCCGGAATAGTAAAGGGGAAAGGCTTCTGCTT : 1355
chimp.fast  : ATGGAATCCATCCAGGGAGAGGCTCGACGGGGTGGCACCATGGAGACTGACGACCATATGGGTGGCATCCCTGCCCGGAATAGTAAAGGGGAAAGGCTTCTGCTT : 2155
hg18_dna    : ATGGAATCCATCCAGGGAGAGGCTCGACGGGGTGGCACCATGGAGACCGATGACCATATGGGTGGCATCCCTGCCCGGAATAGTAAAGGGGAAAGGCTTCTGCTT : 2179
human_pare  : ATGGAATCCATCCAGGGAGAGGCTCGACGGGGTGGTACCATGGAGACTGATGACCATATGGGTGGCATCCCTGCCCGGAATAGTAAAGGGGAAAGGCTTCTGCTT : 1155
chimp_pare  : ATGGAATCCATCCAGGGAGAGGCTCGACGGGGTGGTACCATGGAGACTGATGACCATATGGGTGGCATCCCTGCCCGGAATAGTAAAGGGGAAAGGCTTCTGCTT : 1143
dog_parent  : ATGGAATCCATCCAGGGCGAGGCAAGGCGAGGCGGCACTATGGAGACTGATGACCATATGGGTGGCATCCCTGCCCGTAATAGTAAAGGGGAAAGGCTGCTGCTT : 1152
              ATGGAATCCATCCAGGGaGAGGCtcGacGgGGtGGcACcaTGGAGAC GAtGACCAtATGGGTGGCATCCCTGCCCggAATAGTAAAGGGGAAAGGCTtCTGCTT

                 *      2220         *      2240         *      2260         *      2280         *      2300         *
gibbon.fas  : TATATTGGCATCATTGACATTCTACAGTCTTACAGGTTTGTTAAAAAGTTGGAGCAATCTTGGAAAGCCCTGGTACGTGACGGAGACACTGTCTCAGTGCATCGC : 1957
pongo.fast  : TATATTGGCATCATTGACATTCTACAGTCTTACAGGTTTGTTAAGAAGTTGGAGCACTCTTGGAAAGCCCTGGTACATGATGGAGATACTGTCTCAGTGCATCAC : 2124
gorilla.fa  : TATATTGGCATCATTGACATTCTACAGTCTTACAGGTTTGTTAAGAAGTTGGAGCACTCTTGGAAAGCCCTGGTACATGATGGAGACACTGTCTCAGTGCATCGC : 1460
chimp.fast  : TATATTGGCATCATTGACATTCTACAGTCTTACAGGTTTGTTAAGAAGTTGGAGCACTCTTGGAAAGCCCTGGTACATGACAGAGACACTGTCTCAGTGCATCGC : 2260
hg18_dna    : TATATTGGCATCATTGACATTCTACAGTCTTACAGGTTTGTTAAGAAGTTGGAGCACTCTTGGAAAGCCCTGATACATGACGGAGACACTGTCTCAGTGCATCGC : 2284
human_pare  : TATATTGGCATCATTGACATTCTACAGTCTTACAGGTTTGTTAAGAAGTTGGAGCACTCTTGGAAAGCCCTGGTACATGACGGAGACACTGTCTCAGTGCATCGC : 1260
chimp_pare  : TATATTGGCATCATTGACATTCTACAGTCTTACAGGTTTGTTAAGAAGTTGGAGCACTCTTGGAAAGCCCTGGTACATGACGGAGACACTGTCTCAGTGCATCGC : 1248
dog_parent  : TATATTGGGATCATTGACATTCTCCAGTCTTACAGGTTTGTTAAGAAGTTGGAGCACTCTTGGAAAGCCCTGGTACATGATGGAGACACCGTCTCGGTGCACCGC : 1257
              TATATTGGcATCATTGACATTCTaCAGTCTTACAGGTTTGTTAAgAAGTTGGAGCAcTCTTGGAAAGCCCTGgTACaTGA gGAGAcACtGTCTCaGTGCAtCgC

                   2320         *      2340         *      2360         *      2380         *      2400         *
gibbon.fas  : CCACGCTTCTACGCTAAATGATTCCAGCGCTTCATGTGCAACACAGTATTTAAGGAGATTCCCTTGAAGCCTTCTCCCTACAAAAAGTTTCAGTCTGGCTCATCT : 2062
pongo.fast  : CCAGGCTTCTACGCTGAACGGTTCCAGCGCTTCATCTGCAACACAGTATTTAAGAAGATTCCCTTGAAGCCTTCTCCTTCCAAAAGATTTCGGTCTGGCTCATCT : 2229
gorilla.fa  : CCAGGCTTCTATGCTGAATGGTTCCAGCGCTTCATGTGCAACACAGTATTTAAGAAGATTCCCTTGAAGCCTTCTCCTTCCAAAAAACTTTGGTCTGGCTCATCT : 1565
chimp.fast  : CCAGGCTTCTATGCTGAATGGTTCCAGCGCTTCATGTGCAACACGGTATTTAAGAAGATTCCCTTGAAGCCTTCTCCTTCCAAAAAACTTCAGTCTGGCTCATCT : 2365
hg18_dna    : CCAGGCTTCTATGCTGAATGGTTCCAGCGCTTCATGTGCAACACGGTATTTAAGAAGATTCCCTTGAAGCCTTCTCCTTCCAAAAAACTTCGGTCTGGCTCATCT : 2389
human_pare  : CCAGGCTTCTACGCTGAACGGTTCCAGCGCTTCATGTGCAACACAGTATTTAAGAAGATTCCCTTGAAGCCTTCTCCTTCCAAAAAGTTTCGGTCTGGCTCATCT : 1365
chimp_pare  : CCAGGCTTCTACGCTGAACGGTTCCAGCGCTTCATGTGCAACACAGTATTTAAGAAGATTCCCTTGAAGCCTTCTCCTTCCAAAAAGTTTCGGTCTGGCTCATCT : 1353
dog_parent  : CCAGGCTTCTATGCTGAACGGTTCCAGCGCTTCATGTGCAACACAGTGTTCAAGAAGATACCCTTGAAGCCCTCTCCTTCCAAAAAGTTTCGGTCTGGCTCATCT : 1362
              CCAgGCTTCTA GCTgAA GgTTCCAGCGCTTCATgTGCAACACaGTaTTtAAGaAGATtCCCTTGAAGCCtTCTCCtTcCAAAAa  TTcgGTCTGGCTCATCT

              2420         *      2440         *      2460         *      2480         *      2500         *      2520
gibbon.fas  : TTCTCTCAATGAGCAGGCTCCAGTGGCAACTCCTGCATTATTTACCAGCCACTGGTCTCTGGGGAACACAAGGCACAAGTGACAACAAAGGCGGAAGTGGAGCCA : 2167
pongo.fast  : TTCTCTCAGCGAGCAGGCTCCAGTGGCAACTCCTGCATTACTTACCAGCCATTGGTCTCTGGGGAACACAAGGCACAAGTGACAACAAAGGCGGAAGTGGAGCCA : 2334
gorilla.fa  : TTCTCTCAGCGAGCAGGCTCCAGTGGCAACTCCTGCATGACTTACCAGCCATTGGTCTCTGGGGAACACAAGGCACAAGTGACAACAAAGGCGGAAGTGGAGCCA : 1670
chimp.fast  : TTCTCTCAGCGAGCAGGCTCCAGTGGCAACTCCTGCATTACTTACCAGCCATTGGTCTCTGGGGAACACAAGGCACAAGTGACAACAAAGGCGGAAGTGGAGCCA : 2470
hg18_dna    : TTCTCTCAGCGAGCAGGCTCCAGTGGCAACTCCTGCATTACTTACCAGCCATTGGTCTCTGGGGAACACAAGGCACAAGTGACAACAAAGGCGGAAGTGGAGCCA : 2494
human_pare  : TTCTCTCGGCGAGCAGGCTCCAGTGGCAACTCCTGCATTACTTACCAGCCATCGGTCTCTGGGGAACACAAGGCACAAGTGACAACAAAGGCAGAAGTGGAGCCA : 1470
chimp_pare  : TTCTCTCGGCGAGCAGGCTCCAGTGGCAACTCCTGCATTACTTACCAGCCATCGGTCTCTGGGGAACACAAGGCACAAGTGACAACAAAGGCAGAAGTGGAGCCA : 1458
dog_parent  : TTCTCTCGGCGAGCAGGCCCCAGCGGCAACTCCTGCATTACTTACCAGACATCGGTCTCTGGGGAGCACAAGGCACAAGTGACAACCAAGGCGGAAGTGGAGCCA : 1467
              TTCTCTC gcGAGCAGGCtCCAGtGGCAACTCCTGCATtAcTTACCAGcCAt GGTCTCTGGGGAaCACAAGGCACAAGTGACAACaAAGGCgGAAGTGGAGCCA

                      *      2540         *      2560         *      2580         *      2600         *      2620
gibbon.fas  : GGCGTTCACCTTGGTCGTCCTGATGTTTTACCTCAGATCCCACCTTTGGAGGAAATCAGTGAGGGCTCGCCTATTCCTGACCCCAGTTTCTCACCTCTAGTTGGA : 2272
pongo.fast  : GGCGTTTACCTTGGTCGTCCTGATGTTTTACCTCAGACTCCACCTTTGGAGGAAATCAGTGAGGGCTCACCTATTCCTGATCCCAGTTTCTCACCTCTAGTTGGA : 2439
gorilla.fa  : GGCGTTCACCTTGGTCGTCCTGATGTTTTACCTCAGACTCCACCTTTGGAGGAAATCAGTGAGGGCTCACCTATTCCTGACCCCAGTT----------------- : 1758
chimp.fast  : GGCGTTCACCTTGGTCGTCCTGATGTTTTACCTCAGACTCCACCTTTGGAGGAAATCAGTGAGGGCTCACCTATTCCTGACCCCAGTTTCTCACCTGTAGTTGGA : 2575
hg18_dna    : GGTGTTCACCTTGGTTGTCCTGATGTTTTACCTCAGACTCCACCTTTGGAGGAAATCAGTGAGGGCTCACCTACTCCTGACCCCAGTTTCTCACCTCTAGTTGAA : 2599
human_pare  : GGCGTTCACCTTGGTCGTCCTGATGTTTTACCTCAGACTCCACCTTTGGAGGAAATCAGTGAGGGCTCGCCTATTCCTGACCCCAGTTTCTCACCTCTAGTTGGA : 1575
chimp_pare  : GGCGTTCACCTTGGTCGTCCTGATGTTTTACCTCAGACTCCACCTTTGGAGGAAATCAGTGAGGGCTCGCCTATTCCTGACCCCAGTTTCTCACCTCTAGTTGGA : 1563
dog_parent  : GGCGTCCACTTCGGTCGTCCTGATGTTTTACCTCAGACTCCACCTTTGGAGAAAATCAGTGAGGTTTCGACTATTCCTGACCCTATTTCTCACCTGTAGTTGGA : 1572
              GGcGTtcACcTtGGTcGTCCTGATGTTTTACCTCAGActCCACCTTTGGAGgAAATCAGTGAGGgcTC cCTAtTCCTGAcCCCagTTtctcacct tagttgga

                 *      2640         *      2660         *      2680         *      2700         *      2720         *
gibbon.fas  : GAGACTTTGCAAATGCTAACTACAA---------------------------------------------------------------------------GTGTG : 2302
pongo.fast  : GAGACTTTGCAAATGCTAACTACAA---------------------------------------------------------------------------GTGTG : 2469
gorilla.fa  : ------------------------------------------------------------------------------------------------------- :    -
chimp.fast  : GAGACTTTGCAAACGCTAACTACAA---------------------------------------------------------------------------GTGTG : 2605
hg18_dna    : GAGACTTTGCAAATGCTAACTACAA---------------------------------------------------------------------------GTGTG : 2629
human_pare  : GAGACTTTGCAAATGCTAACTACAAGTACAACCTTGGAAAAGCTTGAAGTTGCAGAGTCAGAGTTCACCCATTAAATGGTGTTGGAAAGCACTATGGTGTGTGTG : 1680
chimp_pare  : GAGACTTTGCAAATGCTAACTACAA---------------------------------------------------------------------------GTGTG : 1593
dog_parent  : GAGACTTTGCCAATGCAAACTACAA---------------------------------------------------------------------------GTGTG : 1602
              gagactttgcaaatgctaactacaa                                                                           gtgtg

                   2740         *      2760         *      2780         *      2800         *      2820         *
gibbon.fas  : GACAACAGTGAGTATATGCAGAATGGAGACTTCTTACCCACCCGGCTGCAGGCCCAGCAGGATGCTGTCAACATAGTTTGTCATTCAAAGACCCGCAGCAACCCT : 2407
pongo.fast  : GACAACAGTGAGTATACGCGGAATGGAGACTTCTTACCCATCCGGCTGCAGGCCCAGCAGGATGCTGTCAACACAGTTTGTCATTCAAAGACCCGCAGCAACCCT : 2574
gorilla.fa  : --------------------------------------------------------------------------------------------------------- :    -
chimp.fast  : GACAACAGTGAGTATATGCGGAATGGAGACTTCTTACCCACCCGGCTGCAGGCCCAGCAGGATGCTGTCAACACAGTTTGTCATTCAAAGACCCGCAGCAACCCT : 2710
hg18_dna    : GACAACAGTGAGTATATGGGGAATGGAGACTTCTTACCCACCCGGCTGCAGGCCCAGCAGGATGCTGTCAACACAGTTTGTCATTCAAAGACCCGCAGCAACCCT : 2734
human_pare  : GACAACAGTGAGTATATGCGGAATGGAGACTTCTTACCCACCAGGCTGCAGGCCCAGCAGGATGCTGTCAACATAGTTTGTCATTCAAAGACCCGCAGCAACCCT : 1785
chimp_pare  : GACAACAGTGAGTATATGCGGAATGGAGACTTCTTGCCCACCAGGCTGCAGGCCCAGCAGGATGCTGTCAACATAGTTTGTCATTCAAAGACCCGCAGCAACCCT : 1698
dog_parent  : GACAACAGTGAGTATATGCGGAATGGGGACTTCTTACCCACCCGGCTGCAGGCCCAACAAGATGCTGTCAACATAGTTTGTCACTCTAAGACCCGCAGCAACCCT : 1707
              gacaacagtgagtatatgcggaatggagacttcttacccacc ggctgcaggcccagcaggatgctgtcaaca agtttgtcattcaaagacccgcagcaaccct

              2840         *      2860         *      2880         *      2900         *      2920         *      2940
gibbon.fas  : GAGAACAACGTGGGCCTTATCACACTGGATAATGACTGTGAAGTGCTGACCACACTCACCCCAGACACTGGCCGTATCCTGTCCAAGCTACATACTGTCCAACCC : 2512
pongo.fast  : GAGAACAACGTGGGCCTTATCACGCTGGATAATGACTGTGAAGTGCTGACCACACTCACCCCAGACACTGGCCGTATCCCGTCCAAGCTACATACTGTCCAACCC : 2679
gorilla.fa  : --------------------------------------------------------------------------------------------------------- :    -
chimp.fast  : GAGAACAACGTGGGCCTTATCACACTGGATAATGACTGTGAAGTGCTGACCACACTCACCCCAGACACTGGCTGTATCCTGTCCAAGCTACATACTGTCCAACCC : 2815
hg18_dna    : GAGAACAACGTGGGCCTTATCACACTGGATAATGACTGTGAAGTGCTGACCACACTCACCCCAGACACTGGCCGTATCCTGTCCAAGCTACATACTGTCCAACCC : 2839
human_pare  : GAGAACAACGTGGGCCTTATCACACTGGCTAATGACTGTGAAGTGCTGACCACACTCACCCCAGACACTGGCCGTATCCTGTCCAAGCTACATACTGTCCAACCC : 1890
chimp_pare  : GAGAACAACGTGGGCCTTATCACACTGGCTAATGACTGTGAAGTGCTGACTACACTCACCCCAGACACTGGCCGTATCCTGTCCAAGCTACATACTGTCCAACCC : 1803
dog_parent  : GAAAACAACGTGGGCCTCATCACACTGGCCAATGACTGTGAAGTGCTGACCACACTCACCCCTGACACTGGCCGCATTCTTTCCAAGCTCCACACTGTGCAACCC : 1812
              gagaacaacgtgggccttatcacactgg taatgactgtgaagtgctgaccacactcaccccagacactggccgtatcctgtccaagctacatactgtccaaccc

                      *      2960         *      2980         *      3000         *      3020         *      3040
gibbon.fas  : AAGGGCAAGAGCACCTTCTGCACGGGCATCAGCGTGGCCCATCTGGCTCTGAAGCGCCGACAAGACAAGAATGACAAGATCCGCATCATTGCCTTTGTGGGAAAC : 2617
pongo.fast  : AAGGGCAAGATCACCTTCTGCACGGGCATCCGCGTGGCCCATCTGGCTCTGAAGCACCGACAAGGCAACAATCACAAGATCCGCATCATTGCCTTTGTGGGAAAC : 2784
gorilla.fa  : --------------------------------------------------------------------------------------------------------- :    -
chimp.fast  : AAGGGCAAGATCACCTTCTGCATGGGCATCCACGTGGCCCATCTGGCTCTGAAGCACCGACAAGGCAACAATCACAAGATCCGCATCATTGCCTTTGTGGGAAAC : 2920
hg18_dna    : AAGGGCAAGATCACCTTCTGCATGGGCATCCACGTGGCCCATCTGGCTCTGAAGCACCGACAAGGCAACAATCACAAGATCCGCATCATTGCCTTTGTGGGAAAC : 2944
human_pare  : AAGGGCAAGATCACCTTCTGCACGGGCATCCGCGTGGCCCATCTGGCTCTGAAGCACCGACAAGGCAAGAATCACAAGATGCGCATCATTGCCTTTGTGGGAAGC : 1995
chimp_pare  : AAGGGCAAGATCACCTTCTGCACGGGCATCCGCGTGGCCCATCTGGCTCTGAAGCACCGACAAGGCAAGAATCACAAGATGCGCATCATTGCCTTTGTGGGAAGC : 1908
dog_parent  : AAGGGCAAGATCACCTTCTGCACTGGTATCCGCGTGGCTCATCTGGCTCTGAAGCACAGGCAGGGCAAGAATCACAAGATGCGCATTATTGCCTTCGTGGGAAGC : 1917
              aagggcaagatcaccttctgca gggcatcc cgtggcccatctggctctgaagcaccgacaaggcaa aatcacaagat cgcatcattgcctttgtgggaa c
```

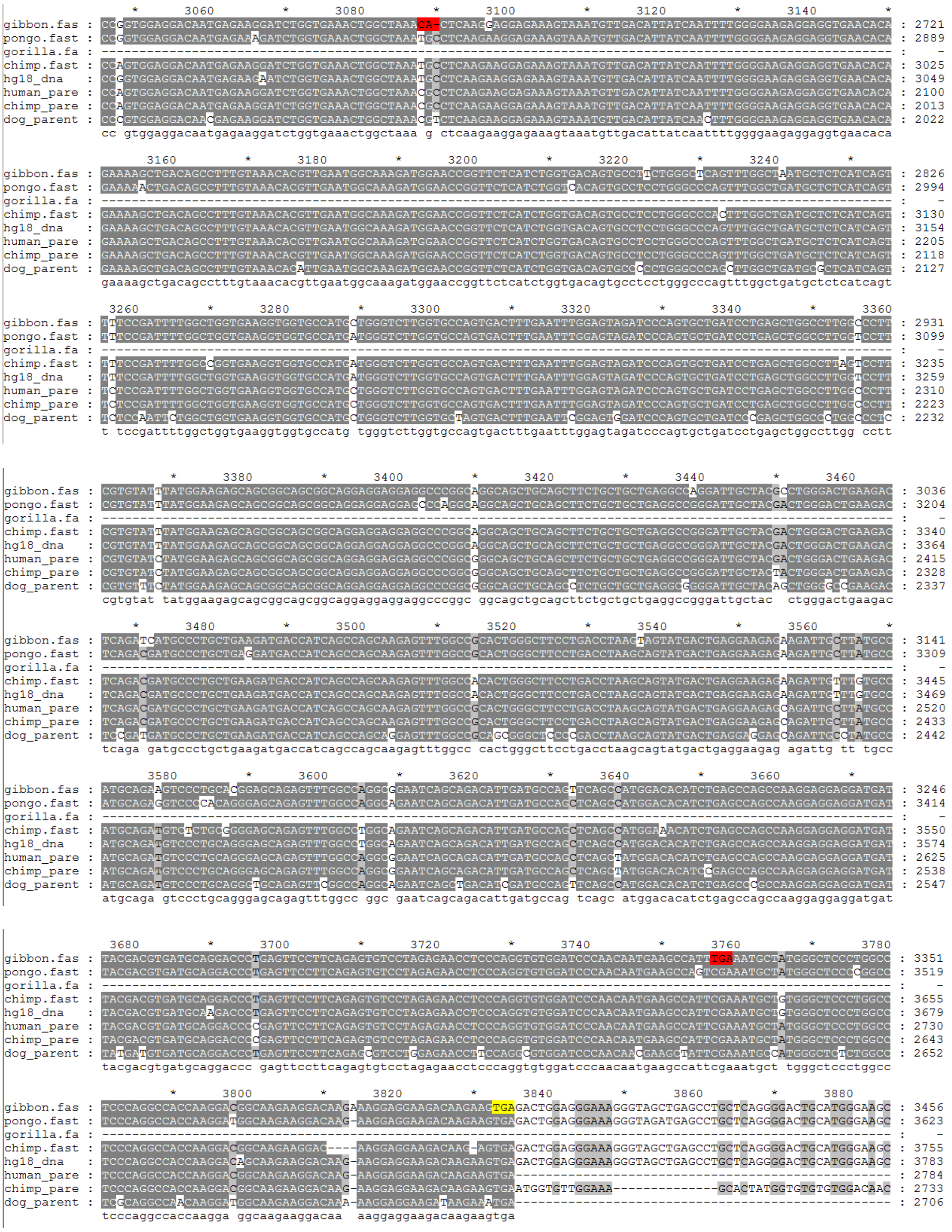

Alignment of the complete *PIPSL* locus across all five all hominoids and the parental genes from human, chimp and dog. The number on top and left indicate the location of nucleotides in alignment and member sequence, respectively. “ATG” and “TGA” in yellow mark the start and stop codon. Nucleotides in red mark the codons in gibbon and gorilla with some disrupting mutations.
