# Supplementary material for: Positive selection for the male functionality of a co-retroposed gene in the hominoids
Source: BMC Evol Biol. 2009 Oct 15;9:252. doi: 10.1186/1471-2148-9-252 (PMC2773790; doi:10.1186/1471-2148-9-252)
Supplement: Additional file 4 — Parameters for likelihood ratio tests in CODEML. Parameters we used in CODEML are summarized. [file 1471-2148-9-252-S4.pdf]

Alignment	Model	Codeml parameters and hypothesis
PIP5K1A-derived region	Branch	*((((((((human,chimp),gorilla),pongo#1),gibbon),<br>(human_p,chimp_p)),rhesus_p),dog_p),opossum_p);*<br>*df=1*<br>*fix_omega = 1*<br>*omega = 1*<br>whether orangutan branch has an omega different from 1<br>*p*=0.020
PSMD4-derived region	Branch	*(((((((human#2,chimp#2)#1,pongo#2)#1,gibbon)#1,<br>(((human_p,chimp_p),pongo_p),gibbon_p)),rhesus_p)<br>,dog_p),opossum_p)*<br>*df=1*<br>whether PSMD4-derived region has two sets of omega for the internal branch and external branch, respectively<br>*p*=0.026
PSMD4-derived region	Branch	*(((((((human,chimp),pongo#1),gibbon),<br>(((human_p,chimp_p),pongo_p),gibbon_p)),rhesus_p)<br>,dog_p),opossum_p)*<br>*fix_omega = 1*<br>*omega = 1*<br>*df=1*<br>whether orangutan branch has an omega different from 1<br>*p*=0.10
PSMD4-derived region	Branch	*(((((((human,chimp)#1,pongo),gibbon),<br>(((human_p,chimp_p),pongo_p),gibbon_p)),rhesus_p)<br>,dog_p),opossum_p)*<br>*fix_omega = 1*<br>*omega = 1*<br>*df=1*<br>whether human/chimp ancestral branch has an omega different from 1<br>*p*=0.033
PSMD4-derived region	Branch	*(((((human,chimp)#1 ,<br>(((human_p,chimp_p),pongo_p),gibbon_p)),rhesus_p)<br>,dog_p),opossum_p)*<br>*fix_omega = 1*<br>*omega = 1*<br>*df=1*<br>whether human/chimp ancestral branch has an omega different from 1<br>*p*=0.043

Alignment	Model	Codeml parameters and hypothesis
PSMD4-derived region	Branch-site	*(((((((human,chimp)#1,pongo),gibbon),(((human_p,chimp_p),pongo_p),gibbon_p)),rhesus_p),dog_p),opossum_p)*<br>*model = 2*<br>*NSsites = 2*
