# Supplementary material for: Positive selection for the male functionality of a co-retroposed gene in the hominoids
Source: BMC Evol Biol. 2009 Oct 15;9:252. doi: 10.1186/1471-2148-9-252 (PMC2773790; doi:10.1186/1471-2148-9-252)
Supplement: Additional file 5 — Alignment of PSMD4-derived region and sites under positive selection detected by HyPhy. For panel A, we showed the alignment of PSMD4-derived region. Yellow bars and red bars marks the mutations occurred in the ancestor of all hominoids and those occurred before the split of human and chimp, respectively. Light blue rectangle define the core residues of UIM-1 (LALALS) and UIM-2 (IAYAMS). The numbers above red bars show the probability calculated by BEB statistics with "*" as a label of significance. For panel B, We used the "SubtreeSelectionComparason" module of HyPhy to infer the sites driven by positive selection on the internal branch, the split of human/chimp and orangutan. The blue and red lines indicate approximate site-specific dN and corresponding Log10-based LRT p, respectively. The yellow line marks the confidence level of p of 0.05. Compared to CODEML, HyPhy identified more sites under adaptive selection. As a remarkable overlap between both methods, UIM-2 is remarkably enriched with such sites. [file 1471-2148-9-252-S5.pdf]

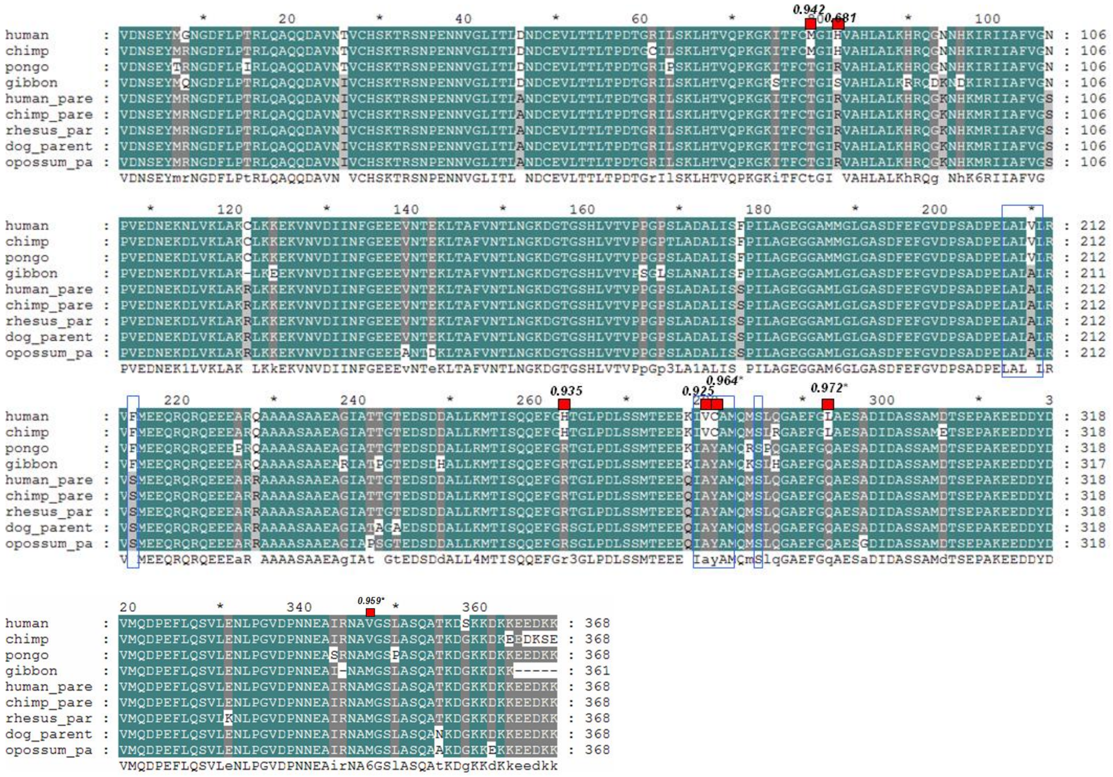

A. Alignments of *PSMD4*-derived region. Yellow bars and red bars marks the mutations occurred in the ancestor of all hominoids and those occurred before the split of human and chimp, respectively. Light blue rectangle define the core residues of UIM-1 (LALAL..S) and UIM-2 (IAYAM..S). The numbers above red bars show the probability calculated by BEB statistics with "*" as a label of significance.

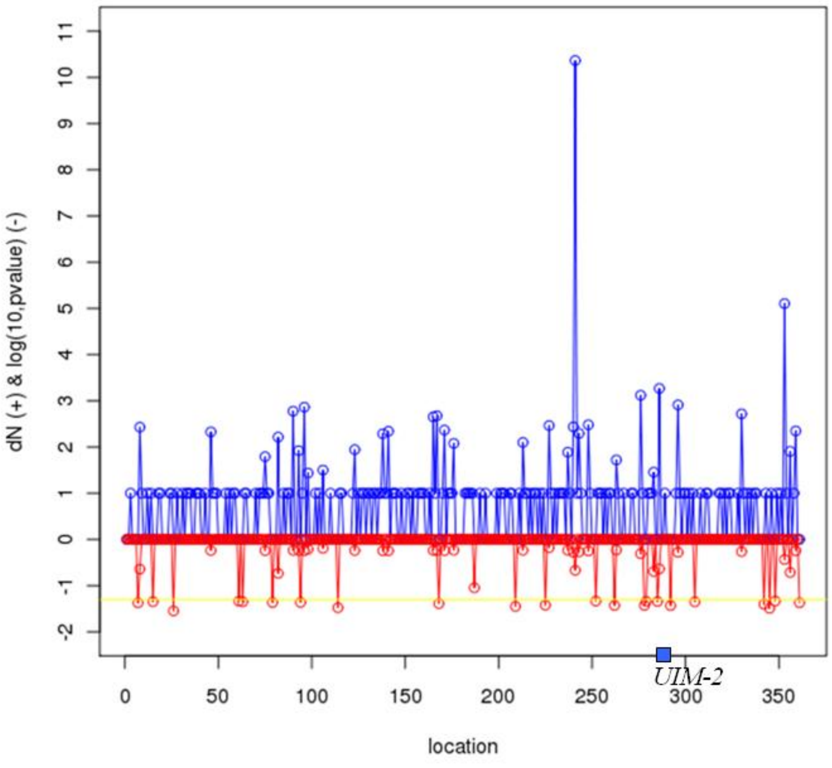

B. We used the "SubtreeSelectionComparason" module of HyPhy to infer the sites driven by positive selection on the internal branch, the split of human/chimp and orangutan. The blue and red lines indicate approximate site-specific *dN* and corresponding Log10-based LRT *p*, respectively. The yellow line marks the confidence level of *p* of 0.05. Compared to CODEML, HyPhy identified more sites under adaptive selection. As a remarkable overlap between both methods, UIM-2 is remarkably enriched with such sites.
