# Supplementary material for: Positive selection for the male functionality of a co-retroposed gene in the hominoids
Source: BMC Evol Biol. 2009 Oct 15;9:252. doi: 10.1186/1471-2148-9-252 (PMC2773790; doi:10.1186/1471-2148-9-252)
Supplement: Additional file 6 — Evolution of PSMD4 across various vertebrates. The pink rectangle marks the PIPSL locus, while the blue bar marks the adaptive evolution of PSMD4 before the split of birds and mammals. The number on all the branches like (#0.0090) shows the Ka/Ks. [file 1471-2148-9-252-S6.pdf]

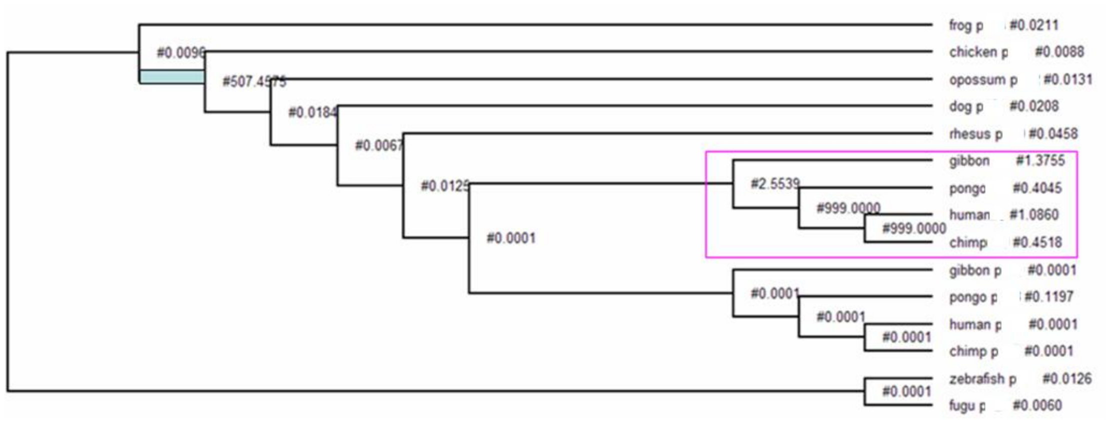

Evolution of PSMD4 across various vertebrate. The pink rectangle marks the PIPSL locus, while the blue bar marks the adaptive evolution of PSMD4 before the split of birds and mammals. The number on all the branches like (#0.0090) shows the *Ka/Ks*.
