# Supplementary material for: Positive selection for the male functionality of a co-retroposed gene in the hominoids
Source: BMC Evol Biol. 2009 Oct 15;9:252. doi: 10.1186/1471-2148-9-252 (PMC2773790; doi:10.1186/1471-2148-9-252)
Supplement: Additional file 7 — Supplementary methods. We described the methodology in identification of retroposed copies and expressional profiling. [file 1471-2148-9-252-S7.pdf]

Supplemental Methods

Identification of retroposed copies

As shown by Supplemental Figure S1 and Table S1 (the end of this file), we integrated previous strategies developed [1-4] to screen RPCs across multiple species (Table S1). 1) Ensembl proteins with status of "Known" or "Novel" are mapped to genomes using TBLASTN [5]. 2) We modified the Pseudopipe [3] package to process the raw alignments in merging BLAST high-score blocks, retaining the best merged hits, inferring the conceptual open reading frame (ORF) of RPCs based on FASTY [6], and predicting polyA tracts. We kept the parameters embedded in Pseudopipe, such as TBLASTN evalue cutoff ($1e^{-10}$) and coverage cutoff (70%), but made the following modifications: to increase identity cutoff from 40% to 50% [7] to discard those un-reliable retrogenes; to correct several bugs possibly caused due to the update of BLAST or FASTA package, to disable masking Ensembl proteins which might cause the missing of annotated Ensembl retrogenes, and to drop the hits mapping to their own genomic loci. 3) Based on BioPerl [8] and Bio-Ensembl Perl scripts [9], we scanned the absence of parental introns according to exon and intron structure information, which map within the alignments between parents and RPCs. In comparison with Marques' strategy [4], we did not exclude all those small introns shorter than 80 bps and we consider polyA tract evidence for those RPCs with less than three intron absent. Small introns with the support of the canonical splicing site (GT-AG) and known non-canonical splicing sites (AT-AC or GC-AG) were also considered, because genuine tiny introns exist [10, 11]. For those RPCs with only one intron absent, we require both the *Ks* smaller than 2 and the presence of polyA tract. For those RPCs with two introns absent but a *Ks* larger than 2, we also require the presence of polyA tract.

After all three steps, we generate a single-exon RPC list. For each RPC, its most similar hit is an Ensembl protein with CDS consisting of multiple exons and at least

one introns missing in the alignments. We further need to detect the protein-coding signatures of RPCs. As shown in the Supplementary Figure, we implemented two independent strategies to generate a reliable but possibly not complete list of retrogene and a complete but possibly false positive containing list of retrogenes.

On the one hand, we used a widely accepted criteria in that Ka/Ks between the parental and retrogene pairs are significantly smaller than 0.5 ($p < 0.05$) calculated by likelihood ratio test. Herein, the codeml program of PAML was used to calculate the ratio. Specifically, we performed two runs, one with ω fixed to 0.5 and another with a free ω. The log likelihood value of the fixed ωmodel (l0) was compared to the free model (li) based on a χ^2 distribution with the degree of freedom as one [12]. Retrogenes were defined as RPCs passing this test. They also need to have intact or nearly intact ORFs, i.e., the largest continuous alignment region without any codon shifts or stop codons covers at least 90% of the whole RPC. Since many RPCs especially those younger or small ones have not enough substitution sites, this strategy only generates a small dataset of retrogenes.

On the other hand, we map the Ensembl annotation to RPCs according to the chromosomal coordinates of Ensembl protein-coding genes. If one RPC overlaps with one Ensembl protein only with status of “Known” (for other species except human and mouse, the status is usually “Known_by_projection”) on the same strand and the conceptual ORF covers at least 70% of this protein, this RPC is thought to have coding potential. Also, we require this ORF is intact or nearly intact. Ensembl seems to annotate many retrogenes. Taking human released on Oct., 2006 as an example, transcripts with only one exon in coding region amount to 5,732. Thus, this strategy pulled out relatively more retrogenes.

Out of 6,750 RPCs, we defined a conservative dataset of 729 retropseudogenes, which meets with all the following creterias: 1) the Ka/Ks (ω) between parental gene and retrogene is larger than 0.5 for a smaller value indicates the functional constraint [1].

2) The conceptual ORF is scattered with frame shifts or stop codons, which render the longest continuous region smaller than 30% of the parental ORF. 3) RPCs do not overlap with any annotated Ensembl proteins [13]. Concurrently, we also generated a less stringent dataset of 5,386 retropseudogenes, which meets with the above first and third criteria. They also consist of at least one frame shift or stop codon but the continuous ORF is larger than 30%.

Expression analysis

We downloaded the normalized expression data of 11 human tissues based on Affymetrix Human Exon 1.0 ST arrays from UCSC [14] and mapped them to pseudogenes based on chromosomal coordinates and strands. Herein, only probe sets with unique chromosomal location were employed and retrogenes mapped to low quality chromosomes (marked by _random) are also discarded. After this filter, 548 out of 729 pseudogenes were retained for the subsequent analysis. For each probe set, we calculated the median across three biological replicates. For each retropseudogene, we defined the expression value as the median of all probe sets.

Other tools

MySQL v5.0.45 (http://www.mysql.com/) is used to handle large-scale data like peptides identified by mass spectrometry, retroposed copies or exon-array data. All statistical analysis is performed on R v2.6.0 [15].

References

1. Emerson JJ, Kaessmann H, Betran E, Long MY: **Extensive gene traffic on the mammalian X chromosome**. *Science* 2004, **303**(5657):537-540.
2. Vinckenbosch N, Dupanloup I, Kaessmann H: **Evolutionary fate of retroposed gene copies in the human genome**. *Proc Natl Acad Sci U S A*

2006, **103**(9):3220-3225.

3. Zhang Z, Carriero N, Zheng D, Karro J, Harrison PM, Gerstein M: **PseudoPipe: an automated pseudogene identification pipeline**. *Bioinformatics* 2006.
4. Marques AC, Dupanloup I, Vinckenbosch N, Reymond A, Kaessmann H: **Emergence of young human genes after a burst of retroposition in primates**. *PLoS biol* 2005, **3**(11):e357.
5. Altschul SF, Madden TL, Schaffer AA, Zhang J, Zhang Z, Miller W, Lipman DJ: **Gapped BLAST and PSI-BLAST: a new generation of protein database search programs**. *Nucleic Acids Research* 1997, **25**(17):3389-3402.
6. Pearson WR, Lipman DJ: **Improved tools for biological sequence comparison**. *Proc Natl Acad Sci U S A* 1988, **85**(8):2444-2448.
7. Dai H, Yoshimatsu TF, Long M: **Retrogene movement within- and between-chromosomes in the evolution of Drosophila genomes**. *Gene* 2006, **385**:96-102.
8. Stajich JE, Block D, Boulez K, Brenner SE, Chervitz SA, Dagdigian C, Fuellen G, Gilbert JG, Korf I, Lapp H *et al*: **The Bioperl toolkit: Perl modules for the life sciences**. *Genome Res* 2002, **12**(10):1611-1618.
9. Stabenau A, McVicker G, Melsopp C, Proctor G, Clamp M, Birney E: **The Ensembl Core Software Libraries**. *Genome Research* 2004, **14**(5):929.
10. Luna C, Hoa NT, Zhang J, Kanzok SM, Brown SE, Imler JL, Knudson DL, Zheng L: **Characterization of three Toll-like genes from mosquito Aedes aegypti**. *Insect Mol Biol* 2003, **12**(1):67-74.
11. Deutsch M, Long M: **Intron-exon structures of eukaryotic model organisms**. *Nucleic Acids Res* 1999, **27**(15):3219-3228.
12. Yang Z: **Likelihood ratio tests for detecting positive selection and application to primate lysozyme evolution**. *Mol Biol Evol* 1998, **15**(5):568-573.
13. Birney E, Andrews D, Caccamo M, Chen Y, Clarke L, Coates G, Cox T, Cunningham F, Curwen V, Cutts T: **Ensembl 2006**. *Nucleic Acids Research*.

14. Kuhn RM, Karolchik D, Zweig AS, Trumbower H, Thomas DJ, Thakkapallayil A, Sugnet CW, Stanke M, Smith KE, Siepel A *et al*: **The UCSC genome browser database: update 2007**. *Nucleic Acids Res* 2007, **35**(Database issue):D668-673.
15. Team RDC: **R: A Language and Environment for Statistical Computing**. *http://wwwR-projectorg* 2007.

Supplemental Tables

Species	Genome version	Genome data source	Ensembl Gene Release
Human	NCBI Build 36.1	UCSC hg18	41 (Oct., 2006)
Mouse	NCBI Build 36	UCSC mm8	41
Rhesus macaque	BCM HGSC v1.0	UCSC rheMac2	41
Dog	Broad Build v2.0	UCSC canFam2	42[a] (Dec., 2006)
Cow	BCM HGSC v2.0	UCSC bosTau2	41
Chimp	NCBI Build 1 Version 1	UCSC panTro2	41
Rat	RGSC 3.4	UCSC RGSC 3.4	41
Opossum	Broad MonDom 4.0	UCSC MonDom 4.0	41
Chicken	WASHUC2	UCSC galGal3	42
D.melanogaster	BDGP 4.3	Ensembl	42[b]

Table S1. Data sources for the genome-wise identification of retroposed copies.

[a]: Dog data were re-downloaded in Dec., 2006 in order to update to the latest genome assembly.

[b]: For the last species, both gene annotation and genome sequences were downloaded from Ensembl, for corresponding tracks of UCSC are some out of date.

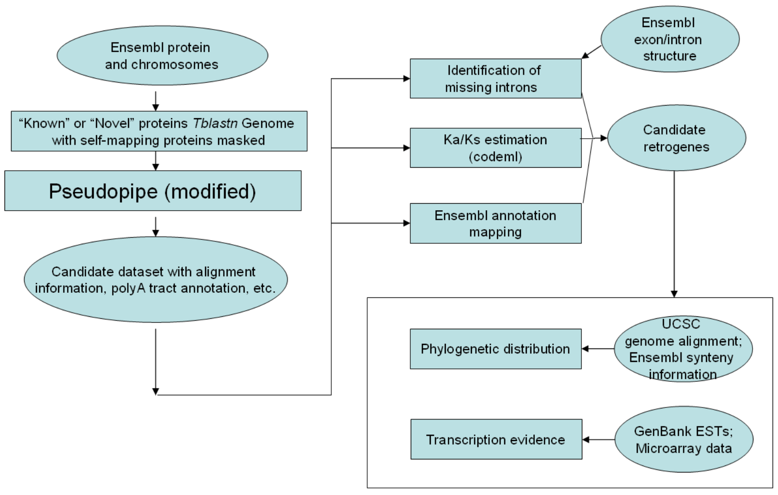

Figure S1. Oval and rectangle indicates the dataset and pipeline, respectively. Here,

the polyA tract in Pseudopipe is defined as a 50 bp window in the 1K 3’ downstream of RPC with at least 30 As.
