# Supplementary material for: Positive selection for the male functionality of a co-retroposed gene in the hominoids
Source: BMC Evol Biol. 2009 Oct 15;9:252. doi: 10.1186/1471-2148-9-252 (PMC2773790; doi:10.1186/1471-2148-9-252)
Supplement: Additional file 8 — Phylogeny of hominoids with estimation of effective population size marked. Numbers in red marks the divergence time in million years, while numbers in black show the estimation of Ne. [file 1471-2148-9-252-S8.pdf]

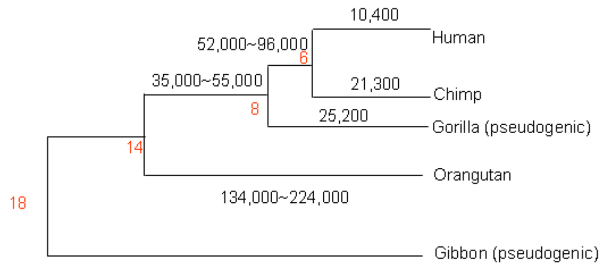

Phylogeny of hominoids. Numbers in red marks the divergence time in million years, while numbers in black show the estimation of *Ne*.
