# Supplementary material for: Positive selection for the male functionality of a co-retroposed gene in the hominoids
Source: BMC Evol Biol. 2009 Oct 15;9:252. doi: 10.1186/1471-2148-9-252 (PMC2773790; doi:10.1186/1471-2148-9-252)
Supplement: Additional file 9 — A similar retroposed fused locus in mouse. 6030436E02Rik and C330019G07Rik encoded by chromosome 5 were co-retroposed to chromosome 8. [file 1471-2148-9-252-S9.pdf]

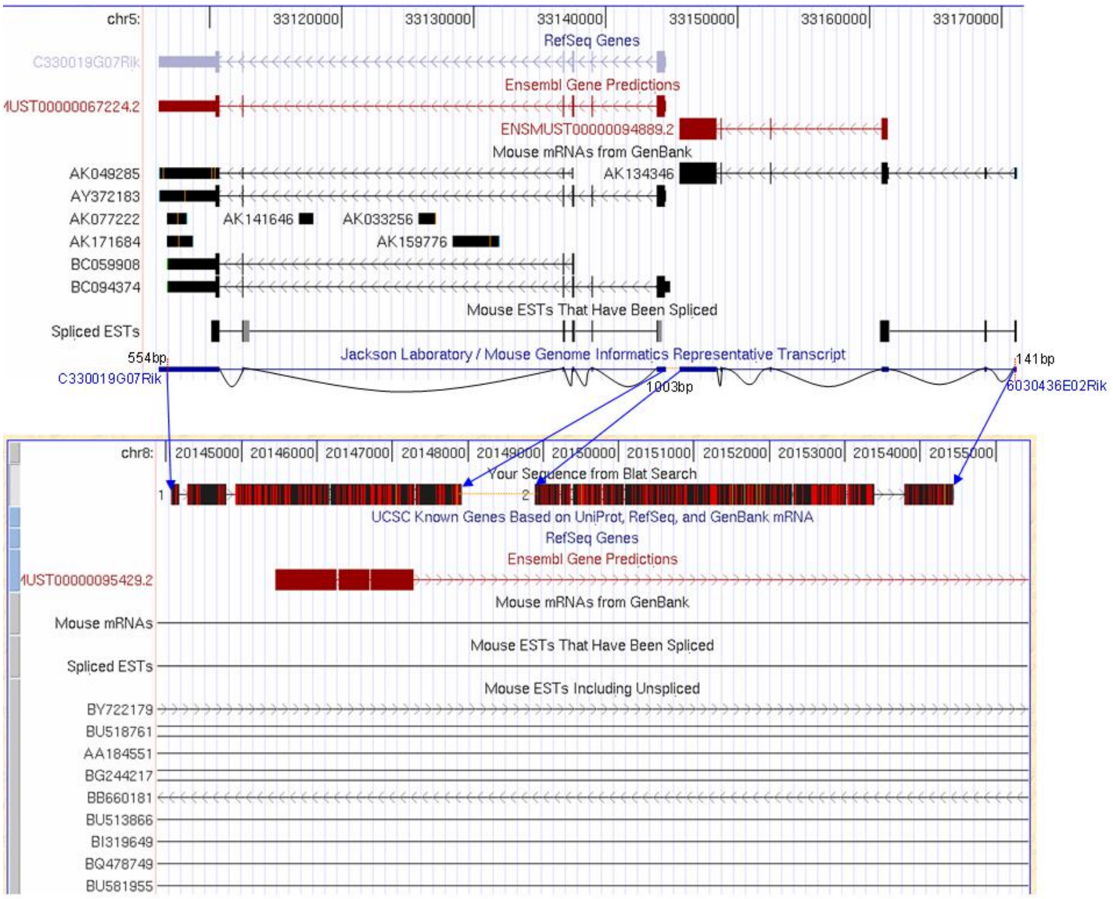

A similar retroposed fused locus in mouse. According to UniGene database [1], both parental genes have a wide expression across blood, brain, pancreas, testis and so on. 6030436E02Rik is inferred based on reviewed computer annotation to have electron carrier activity and heme binding function [2]. As for C330019G07Rik, it is named as embryo implantation factor 2 (EMO2), which might be important for mouse embryo implantation process [3]. This locus emerged after the split of mouse and rat according to genomic alignment information of UCSC [4]. It consists of almost the full-length transcripts of both parental genes except one 141bp region in 5’ UTR of 6030436E02Rik and one 554bp region in 3’ UTR of C330019G07Rik. Moreover, the intergenic 1003bp was also included. Compared to *PIPSL*, inclusion of this genomic DNA region and 5’ UTR of C330019G07Rik might interrupt the continuity of the fused ORF.

1. Wheeler DL, Barrett T, Benson DA, Bryant SH, Canese K, Chetvernin V, Church DM, Dicuccio M, Edgar R, Federhen S *et al*: **Database resources of the National Center for Biotechnology Information**. *Nucleic Acids Res* 2008, **36**(Database issue):D13-21.
2. Carninci P, Kasukawa T, Katayama S, Gough J, Frith MC, Maeda N, Oyama R, Ravasi T, Lenhard B, Wells C: **The Transcriptional Landscape of the Mammalian Genome**. In., vol. 309: American Association for the Advancement of Science; 2005: 1559-1563.
3. Sun Z, Su R, Yang Z, Shi H, Liu C, Wang J: **Expression of the novel gene embryo implantation factor 2 (EMO2) in the mouse uterus at the implantation sites**. *Fertil Steril* 2008.
4. Schwartz S, Kent WJ, Smit A, Zhang Z, Baertsch R, Hardison RC, Haussler D, Miller W:

Human-Mouse Alignments with BLASTZ. *Genome Research* 2003, **13**(1):103-107.
